# Multipathogen Analysis of IgA and IgG Antigen Specificity for Selected Pathogens in Milk Produced by Women From Diverse Geographical Regions: The INSPIRE Study

**DOI:** 10.3389/fimmu.2020.614372

**Published:** 2021-02-11

**Authors:** Michelle K. McGuire, Arlo Z. Randall, Antti E. Seppo, Kirsi M. Järvinen, Courtney L. Meehan, Debela Gindola, Janet E. Williams, Daniel W. Sellen, Elizabeth W. Kamau-Mbuthia, Egidioh W. Kamundia, Samwel Mbugua, Sophie E. Moore, Andrew M. Prentice, James A. Foster, Gloria E. Otoo, Juan M. Rodríguez, Rossina G. Pareja, Lars Bode, Mark A. McGuire, Joseph J. Campo

**Affiliations:** ^1^ Margaret Ritchie School of Family and Consumer Sciences, University of Idaho, Moscow, ID, United States; ^2^ Antigen Discovery Incorporated, Irvine, CA, United States; ^3^ Department of Pediatrics, University of Rochester, Rochester, NY, United States; ^4^ Department of Anthropology, Washington State University, Pullman, WA, United States; ^5^ Department of Anthropology, Hawassa University, Awasa, Ethiopia; ^6^ Department of Animal and Veterinary Science, University of Idaho, Moscow, ID, United States; ^7^ Dalla Lana School of Public Health, University of Toronto, Toronto, ON, Canada; ^8^ Department of Human Nutrition, Egerton University, Nakuru, Kenya; ^9^ Department of Women and Children’s Health, King’s College London, London, United Kingdom; ^10^ MRC Unit, The Gambia at the London School of Hygiene and Tropical Medicine, Banjul, Gambia; ^11^ Department of Biological Sciences, University of Idaho, Moscow, ID, United States; ^12^ Department of Nutrition and Food Science, University of Ghana, Accra, Ghana; ^13^ Department of Nutrition and Food Science, Complutense University of Madrid, Madrid, Spain; ^14^ Nutrition Research Institute, Lima, Peru; ^15^ Larsson-Rosenquist Foundation Mother-Milk-Infant Center of Research Excellence, University of California, San Diego, La Jolla, CA, United States; ^16^ Department of Pediatrics, University of California, San Diego, La Jolla, CA, United States

**Keywords:** human milk, immunoglobulins, IgA, IgG, pathogen, protein array, breastmilk, breastfeeding

## Abstract

Breastfeeding provides defense against infectious disease during early life. The mechanisms underlying this protection are complex but likely include the vast array of immune cells and components, such as immunoglobulins, in milk. Simply characterizing the concentrations of these bioactives, however, provides only limited information regarding their potential relationships with disease risk in the recipient infant. Rather, understanding pathogen and antigen specificity profiles of milk-borne immunoglobulins might lead to a more complete understanding of how maternal immunity impacts infant health and wellbeing. Milk produced by women living in 11 geographically dispersed populations was applied to a protein microarray containing antigens from 16 pathogens, including diarrheagenic *E. coli*, *Shigella* spp.*, Salmonella enterica* serovar Typhi, *Staphylococcus aureus*, *Streptococcus pneumoniae, Mycobacterium tuberculosis* and other pathogens of global health concern, and specific IgA and IgG binding was measured. Our analysis identified novel disease-specific antigen responses and suggests that some IgA and IgG responses vary substantially within and among populations. Patterns of antibody reactivity analyzed by principal component analysis and differential reactivity analysis were associated with either lower-to-middle-income countries (LMICs) or high-income countries (HICs). Antibody levels were generally higher in LMICs than HICs, particularly for *Shigella* and diarrheagenic *E. coli* antigens, although sets of *S. aureus*, *S. pneumoniae*, and some *M. tuberculosis* antigens were more reactive in HICs. Differential responses were typically specific to canonical immunodominant antigens, but a set of nondifferential but highly reactive antibodies were specific to antigens possibly universally recognized by antibodies in human milk. This approach provides a promising means to understand how breastfeeding and human milk protect (or do not protect) infants from environmentally relevant pathogens. Furthermore, this approach might lead to interventions to boost population-specific immunity in at-risk breastfeeding mothers and their infants.

## Introduction

Human milk is the gold standard for infant nutrition, particularly during the first 6 months of life (1, 2). Although geography, socioeconomic factors, resources, and myriad other factors modify benefits of breastfeeding, infants who are fed human milk have lower risks for many serious childhood illnesses including infectious diarrheal and respiratory diseases (3, 4). Breastfeeding is also associated with lower risks of long-term outcomes such as obesity, diabetes, and cardiovascular disease, particularly in preterm infants (5–7). How breastfeeding protects against these diseases and conditions is multifactorial, but human milk’s complex milieu of nutrients, cells, and other biologically active factors likely plays a major role.

For instance, human milk contains complex carbohydrates (human milk oligosaccharides, HMOs) thought to shape the infant’s gastrointestinal (GI) microbial community structures in such a way that harmful taxa are excluded and/or removed (8, 9). Human milk also contains a battery of biologically-active compounds such as fatty acids, lactoferrin, haptocorrin, and α_1_-antitrypsin thought to decrease the risk of infant infection (10, 11). Human milk also harbors a unique and rich bacterial community, which may function in concert with its array of prebiotic and antimicrobial components to shape the recipient infant’s GI and respiratory microbiomes (12–16). Maternally derived immune factors and immune cells, many of which have been primed to fend off the pathogens endemic to an infant’s environment, are most certainly important in this regard (17, 18). These factors include aspects of both innate and acquired immune systems, such as neutrophils, B and T cells, natural killer cells, cytokines, chemokines, complement system components, and immunoglobulins. The immunoglobulins, most of which are present as secretory immunoglobulin A (IgA), are thought to be directed at pathogens with which the mother has had contact, representing a form of dynamic immunological memory (19).

Whereas only a small proportion of the IgA consumed by breastfeeding infants is absorbed, its function is thought to be a first-line defense against foreign antigens present within the newborn’s intestine (20). For instance, IgA in colostrum has been shown to react to enteropathogenic *Escherichia coli* (EPEC) antigens in the feces of breastfed infants, likely important to the protective effect of breastfeeding on diarrheagenic *E. coli* strains (21, 22). In another study, the concentration of anti-*Giardia lamblia* IgA in human milk consumed by infected but asymptomatic infants living in a low-income Mexico City neighborhood, was higher than that of human milk consumed by infected and symptomatic infants living in the same area (23). This suggests a protective effect of these antibodies, at least in terms of symptomatology. Other studies have demonstrated relatively high levels of IgA to *Shigella* plasmid-coded virulence antigens in milk produced by women currently living in areas with both high- and low-risk for *Shigella* infection (Mexico City and Houston, respectively) (24, 25). They suggested that the frequency and persistence of these antibodies targeted toward *Shigella* in the milk of Houston women point to the possibility that a woman’s “memory and drive for secretion of these antibodies is extremely long lived” – a conclusion also reached by Ciardelli and colleagues who studied *E. coli*-specific IgA in milk produced by women who immigrated from high-risk regions but who currently lived in a low-risk region of northern Italy (26). Hayani et al. also reported that concentration of anti-*Shigella* IgA to virulence plasmid-associated antigens in human milk was 8-fold higher in mothers of healthy (yet high-risk) infants compared to those mothers with infants living in the same region but developing diarrhea (27). Collectively, these and other studies point to the likely importance of specific antibodies in milk potentially customized toward protecting a breastfed infant from historically endemic pathogens. However, aside from a few studies designed to characterize immunoglobulin specificity using limited antigenic targets, little is known about the complex immunoglobulin profiles found in human milk in terms of relative specificities to the multiple pathogens encountered by nursing mothers and their recipient infants.

One method of gaining insight as to the specificity of milk-borne immunoglobulins is the use of high-throughput protein arrays featuring multiple antigens associated with selected pathogens. This approach has been used with serum to characterize specific immune responses to selected pathogens and their associated antigens in humans, agricultural animals, and preclinical models (28–30). The method, however, has not been used to characterize the complex immune specificity to multiple pathogens in any sample type, nor has it been used for human milk. Being able to describe the specific and complex immunoglobulin milieu of a woman’s milk will offer useful insight as to how breastfeeding protects infants living in high-risk environments from illness.

Here, we first explored whether high-throughput, protein array methodologies used previously with other biological specimens could be modified to characterize complex immune specificities of IgA and IgG in milk. We then expanded the method to simultaneously consider multiple pathogens rather than a single pathogen. In particular, we were interested in pathogens (and groups, thereof) known to cause neonatal diarrheal and respiratory diseases in the world’s poorest regions. These included EPEC, enterotoxigenic *E. coli* (ETEC), enteroaggregative *E. coli* (EAEC), *Shigella* spp.*, Salmonella enterica* serovar Typhi, *Staphylococcus aureus*, *Streptococcus pneumoniae*, and *Mycobacterium tuberculosis*. Having determined that the methods could indeed be used for the milk matrix (see Results), we conducted exploratory analyses to compare and contrast relative IgA and IgG reactivities to selected antigens known to reflect enhanced responsivity in other biological sample types. Importantly, milk samples analyzed in this study were obtained from healthy, breastfeeding women living in 11 locations around the world. Previous analysis of these samples indicated that concentrations of immunoglobulins, cytokines, and chemokines varied among cohorts, making the samples particularly relevant to the present proof-of-concept study (31). Our overarching hypotheses were that IgA and IgG specificity would vary by pathogen and cohort and be generally higher in low-to-middle income countries (LMIC) compared to high-income countries (HIC) as classified by the World Health Organization (32).

## Material and Methods

### Experimental Design, Subjects, and Ethics Approvals

This investigation was conducted as part of the “INSPIRE” study designed to characterize and compare complex carbohydrate and microbial community structure in milk produced by relatively healthy women around the globe. A detailed description of the study as well as initial findings and participant characteristics have been published elsewhere (16, 31, 33). Briefly, women had to be healthy; breastfeeding or pumping at least five times daily; self-reported-healthy and nursing relatively healthy infants; ≥ 18 years of age; and generally between 1 and 3 months postpartum. Our 11 cohorts were drawn from eight countries: rural and urban Ethiopia (ETR and ETU, respectively), rural and urban The Gambia (GBR and GBU, respectively), Ghana (GN), Kenya (KE), Peru (PE), Spain (SP), Sweden (SW) and the United States [Washington (USW) and California (USC)]. Ethics approvals were obtained for all procedures from each participating institution, with overarching approval from the Washington State University Institutional Review Board (#13264). A total of 413 women were enrolled in the study, and milk was collected from 412 of them. Here, we report data obtained from milk collected from 404 women as follows: ETR (*n* = 38), ETU (*n* = 40), GBR (*n* = 40), GBU (*n* = 40), GN (*n* = 40), KE (*n* = 42), PE (*n* = 43), SP (*n* = 41), SW (*n* = 24), USC (*n* = 15), USW (*n* = 41).

### Milk Collection and Preservation

In PE, SW, USC, and USW, milk was collected using an electric breast pump. In all other cohorts, milk was manually expressed. Except for those collected in ETR, samples were immediately placed on ice, aliquoted within 30 min, and frozen at −20°C. Milk collected in ETR was preserved in a 1:1 ratio with Milk Preservation Solution (Norgen Biotek, Cat. 44800, Thorold, Ontario) and frozen within 6 d. We have shown previously that this method can maintain bacterial DNA integrity in human milk held at 37°C for at least 2 weeks (34). All samples were shipped on dry ice to the University of Idaho, where they were immediately frozen at −20°C.

### Optimization of Protein Array Methodology for Human Milk

Methods used to prepare the milk for protein array analysis were adapted from those of Espinosa-Martos et al (35). Briefly, after being thawed on ice and homogenized, whole milk was centrifuged for 15 min at 30,000xg at 4°C. Avoiding both the fat layer and cell pellet, the skim layer was then carefully removed. This procedure was repeated on the skim fraction, and the resultant supernatant refrozen at −20°C until it was analyzed for its immune specificity.

### Selection of Antigens for Protein Arrays

The multipathogen array was designed to include approximately 80 proteins from each primary focus gastrointestinal, respiratory, and sepsis-related pathogen, as well as 5–15 proteins from secondary pathogens relevant to global health. Selection of proteins followed two approaches: 1) an empirical approach utilizing the databases from prior studies performed at Antigen Discovery, Inc. (ADI, Irvine, CA), and 2) a hypothetical approach using *in silico* prediction of antigenic targets and orthologues of confirmed antigenic targets already identified in ADI databases. Proteins were selected for inclusion based on seroprevalence rates and correlation with exposure to pathogens, or where limited data were available, homology with other antigens. Recombinant purified proteins were included based on commercial availability or provision through partners and collaborators. A detailed explanation of protein selection can be found in the **Supplemental Information**.

### Proteome Microarray Construction

Proteome microarrays were fabricated as previously described using a library of partial or complete CDSs cloned into a T7 expression vector pXI that has been established previously at Antigen Discovery, Inc (36). Briefly, the clone library was created through an *in vivo* recombination cloning process with PCR-amplified coding sequences, and a complementary linearized expressed vector transformed into chemically competent *E. coli* cells was amplified by PCR and cloned into the pXI vector using a high-throughput PCR recombination cloning method as described in detail elsewhere (37). All 783 clones were sequenced (Retrogen, Inc., San Diego, CA), and the results matched the correct target for all clones. Proteins were expressed using an *E. coli in vitro* transcription and translation (IVTT) system (Rapid Translation System, 5 Prime, Gaithersburg, MD, USA; currently provided by biotechrabbit GmbH, Cat. BR1400102, Hennigsdorf, Germany). Each expressed protein includes a 5’ polyhistidine (His) epitope tag and a 3’ hemagglutinin (HA) epitope tag. After expressing the proteins according to the manufacturer’s instructions, translated proteins were printed onto nitrocellulose-coated glass AVID slides (Grace Bio-Labs, Inc., Bend, OR) using an OmniGrid accent robotic microarray printer (Digilabs, Inc., Marlborough, MA). Each slide contained eight nitrocellulose pads on which the full array was printed (this allowed eight samples to be probed per slide using sealed chambers that isolate the arrays). In addition to the targeted proteins, IVTT reactions without expression insert were included and spotted in replicates on each subarray of each pad. These “IVTT controls” served as a normalization factor for array-to-array variation. At approximately 1 nL deposition per spot, IVTT protein per spot was estimated to range between 0.04-0.4ng according to manufacturer guidance. No normalization was done for variation in amount of protein deposited between spots; intra-sample between-protein analysis was not planned. Microarray chip printing and protein expression were quality checked by probing random slides with anti-His and anti-HA monoclonal antibodies with fluorescent labeling. Consistency in protein deposition between slides allows for valid intersample comparisons at the protein level.

### Probing Details

Milk samples were diluted 1:5 in a 1.5 mg/ml *E. coli* lysate solution (Antigen Discovery, Inc., Irvine, CA) in protein arraying buffer (GVS, Cat. 10485356, Sanford, ME) and incubated at room temperature for 30 min. For milk samples previously diluted in preservative solution, samples were diluted 1:2.5 in the lysate solution. Microarray slides were hydrated in arraying buffer and then probed with 250 µl of the preincubated milk samples using sealed, fitted slide chambers to avoid cross-contamination between arrays. Arrays were incubated overnight at 4°C with agitation, washed three times with Tris-buffered saline (TBS)-0.05% Tween 20 (Thermo Scientific, J77500K8, diluted 20x in molecular grade water), and incubated with Cy3-conjugated anti-human IgG diluted 1:200 in arraying buffer and biotin-conjugated anti-Human IgA diluted 1:1000 (Jackson ImmunoResearch, Cat. 709-165-098 and Cat. 109-065-011, West Grove, PA). Arrays were washed three times with TBS–0.05% Tween 20 and incubated with streptavidin-conjugated SureLight P-3 (Columbia Biosciences, Cat. D7-2212, Frederick, MD) at room temperature, protected from light. Arrays were washed 3x with TBS–0.05% Tween 20, 3x with TBS, and once with water and then air dried by being centrifuged at 1,000 x g for 4 min and left overnight in a desiccator before scanning. Probed microarrays were scanned using a GenePix 4,300 A high-resolution microarray scanner (Molecular Devices, Sunnyvale, CA), and an image file (.tiff) was saved for each array using GenePix pro 7 software. The signals in the scanned images were quantified using Mapix software (Innopsys). All further data processing was performed in R (http://www.R-project.org). Data were normalized by first transforming raw values using the base 2 logarithm. Next, the data set was normalized to remove systematic effects by subtracting the median signal intensity of the IVTT control spots for each sample. Since the IVTT control spots carry not only the chip, sample, and batch-level systematic effects, but also antibody background reactivity to the *E. coli* cell-free IVTT system, this procedure normalizes the data and provides a relative measure of the specific antibody binding versus the nonspecific antibody binding to the IVTT controls. With the normalized data, a value of 0.0 means that the intensity is no different than that of the IVTT controls, a value of 1.0 indicates a doubling (2-fold) with respect to IVTT control spots, a value of 2.0 indicates 4-fold, 3.0 indicates 8-fold, etc. IgA and IgG normalized data were analyzed separately, due to independent normalized intensity scales.

### Bioinformatics and Statistics

An *a priori* statistical analysis plan was drafted and reviewed prior to data acquisition and preprocessing. Additional details and *post hoc* analytical procedures are included in the **Supplemental Information**. Briefly, protein seropositivity was defined for each antigen as twice the sample-specific (array-level) median IVTT background, or a normalized signal of 1.0. Antibody breadth was calculated as the number of seropositive protein responses for an individual sample. IgA and IgG breadths were represented on a per-pathogen basis as antibody “breadth score,” calculated as the sum of seropositive responses per pathogen divided by the total number of probes for the corresponding pathogen, i.e. the proportion of positive probes. Summary statistics included mean and median breadth scores, and 95% confidence intervals and interquartile ranges. Antibody magnitude was analyzed as the continuous normalized signal intensity data. Both breadth and magnitude were assessed for differential reactivity between the study cohorts using ANOVA. Each pair of cohorts was tested using T-tests to construct a P-value matrix of pairwise differences. To assess factors associated with IgA and IgG reactivity, multivariable linear regression models were fit with normalized signal intensity of individual protein responses as the dependent variables, that included the cohorts as a categorical predictor variable and the following covariables: mother’s age (y), sex of the breastfeeding infant (male/female), time postpartum (d), parity (# of births), delivery method (cesarean/vaginal), body mass index (BMI) classification (under-, normal-, over-, and obese-weight), mother’s height (cm), presence or absence of companion animals or agricultural animals, and household density [(# of individuals residing in the home) ÷ (# of bedrooms + 1); note that we added +1 to all to adjust for living/bedroom space homes without bedrooms]. All P-values were adjusted for the false discovery rate (38).

## Results and Discussion

### Sample Dilution and Nonspecific Binding

The microarray assay was performed as a dual-labeling experiment, where the Cy3 (green) channel was used to measure IgG responses and the Cy5 (red) channel was used to measure IgA responses. This necessitated using a single sample dilution for the experiments (1:2.5 for milk with preservative and 1:5 for milk without preservative). As described previously, milk collected in rural Ethiopia was chemically preserved because there did not exist reliable electricity in this location (33). The dilution was decided upon by assessing signal-to-noise ratio of the most reactive spots and the number of reactive spots for both IgG and IgA. Due to the restriction of performing IgA and IgG assays simultaneously, the sample dilution had to be balanced to allow detection of the lower concentrations of IgG and the markedly higher concentrations of IgA (31). A greater dilution of samples could be used if there had only been interest in IgA. This technical point is raised because the obtained data showed many IgA responses across all cohorts to antigens from pathogens where there is likely a low risk of exposure. This antibody binding is in part likely cross-reactive and/or polyspecific and might be reduced with more diluted samples. This technical factor may be mitigated by the normalization procedure applied to the data, which accounts for IgA reactivity against components of the *E. coli* cell-free expression system. Despite the observed polyspecific binding, strong IgA responses with differential reactivity between cohorts could be observed with the assay conditions.

### Antigen Down-Selection From Complete Proteomes

A primary set of pathogens (ETEC, EPEC, EAEC, *Shigella* spp., *Salmonella enterica* Typhi, *Staphylococcus aureus*, *Streptococcus pneumoniae*, and *Mycobacterium tuberculosis*) was selected for inclusion on the protein microarray with a target of approximately 80 proteins per pathogen. A secondary set of pathogens (*P. falciparum*, *O. volvulus*, measles virus, rubella virus, dengue virus, Zika virus, yellow fever virus, and chikungunya virus) was used in the microarray with up to 15 proteins per pathogen. Empirical data from prior experiments performed with serum assayed on full or partial proteome protein microarrays were used for selection of proteins with evidence of reactivity with antibodies and association with pathogen infection, history of exposure and protection from the pathogen (details in **Supplemental Information**).

### IgA and IgG Specific to Endemic Pathogens Can Be Detected in Human Milk

Immunoglobulins specific to ETEC, *S. aureus*, and *S. enterica* Typhi were first detected in two human milk samples probed on full proteome and down-selected, single-pathogen microarrays containing cell-free *in vitro* transcription and translation (IVTT) expressed proteins. The dilution factors were optimized separately for both fresh milk samples and those stored in preservative. The numbers of reactive proteins and IgA and IgG signal levels of ETEC and *Salmonella* proteins on the microarrays for the two probed samples correlated with whole anti-bacteria IgA1 ELISA optical density measurements for *E. coli* and *Salmonella typhimurium* (**Figure S1**). *S. aureus* whole bacteria ELISA was not performed, but *Enterococcus faecalis*, *Lactobacillus reuteri*, *Streptococcus equi*, *Enterobacter cloacae*, and *Morganella morganii* ELISA were performed (prior to protein microarray experiments, unpublished data) and showed agreement with *S. aureus* IgA responses by protein microarray, sample JL112015 being the more reactive sample—*S. aureus* protein microarray IgG responses were similar between the two samples tested (**Figures S1B, D**). Additionally, blocking buffers with and without BSA and secreted vs. total IgA were tested. Correlations were high and CVs low in all comparisons (**Figure S1E**). Probing the milk samples on the multipathogen array demonstrated specific antibody binding against numerous proteins from the primary set of pathogens, and at least some reactivity against proteins from the secondary set of pathogens (**Figure S2**).

### Distribution of IgA and IgG Profiles Varied by Pathogen and Ig Type

**Figure 1:**
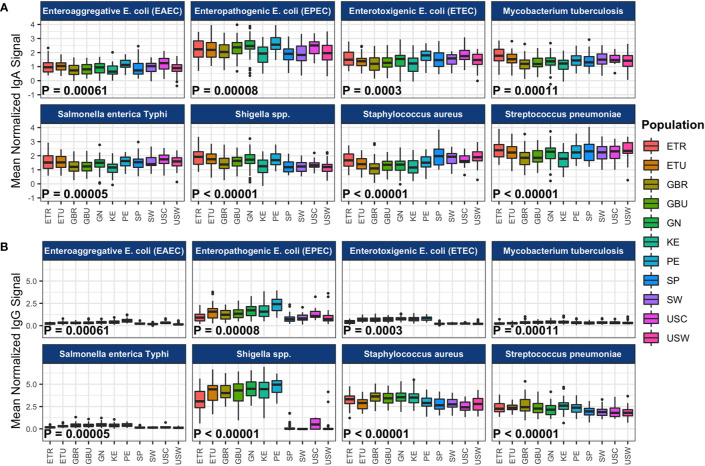
Population-level immunoglobulin A (IgA) and immunoglobulin G (IgG) reactivity to specific pathogen proteins. The boxplots represent the distributions of individuals’ mean normalized **(A)** IgA and **(B)** IgG signal intensities for the 10 most reactive proteins per pathogen, for the primary pathogens. Reactivity was determined by the mean signal intensity per protein across all samples in the study. Samples from each cohort are displayed in colored boxes. Normalized signal intensity scales for IgA and IgG are independent. Abbreviations: ETR, rural Ethiopia; ETU, urban Ethiopia; GBR, rural The Gambia; GBU, urban The Gambia; GN, Ghana; KE, Kenya; PE, Peru; SP, Spain; SW, Sweden; USC, U.S.-California; USW, U.S.-Washington.

The effect of cohort on average normalized signals to the 10 antigens most reactive to IgA or IgG for each primary pathogen is illustrated in **Figure 1** (data provided in **Table 1**), and pairwise cohort comparisons of pathogen-specific IgA and IgG means are shown in **Tables S1A, B**. IgA responses tended to be widely distributed, with responses seen for most pathogens in all populations, with the exception of measles virus, rubella virus, the arboviruses, and *O. volvulus*, which had most signals at or near background levels (**Figure 1A**, **Table S1A**). For overall IgA reactivity, there was an effect of cohort for ETEC, EPEC, EAEC, *Shigella*, *S. enterica* Typhi*, S. aureus*, *S. pneumoniae*, *M. tuberculosis*, *P. falciparum*, measles virus, and Zika virus. For instance, IgA reactivity to *Shigella* was higher in Peru than in Spain (*P_adj_* < 0.001, **Table S1A**).

**Table 1:** Immunoglobulin A (IgA) breadth score and mean [SEM] reactivities to the 10 most-reactive antigens, and mean reactivities to all antigens for each of the primary pathogens included on the protein array in milk produced by women living in rural Ethiopia (ETR), urban Ethiopia (ETU), rural The Gambia (GBR), urban The Gambia (GBU), Ghana (GN), Kenya (KE), Peru (PE), Spain (SP), Sweden (SW), US-California (USC), and US-Washington (USW).

Pathogen	Sig	ETR	ETU	GBR	GBU	GN	KE	PE	SP	SW	USC	USW
**ETEC**												
Breadth score	***	0.21 [0.01]	0.21 [0.01]	0.16 [0.02]	0.17 [0.01]	0.19 [0.01]	0.15 [0.02]	0.24 [0.01]	0.2 [0.02]	0.21 [0.02]	0.27 [0.02]	0.2 [0.01]
Top 10 antigens	***	1.55 [0.1]	1.41 [0.08]	1.26 [0.09]	1.32 [0.09]	1.47 [0.1]	1.18 [0.09]	1.79 [0.07]	1.53 [0.1]	1.48 [0.1]	1.82 [0.15]	1.48 [0.08]
All antigens	**	0.45 [0.04]	0.48 [0.04]	0.33 [0.04]	0.38 [0.04]	0.39 [0.04]	0.31 [0.04]	0.53 [0.04]	0.43 [0.05]	0.46 [0.04]	0.59 [0.06]	0.41 [0.04]
**EPEC**												
Breadth score	***	0.15 [0.01]	0.17 [0.01]	0.15 [0.01]	0.16 [0.01]	0.17 [0.01]	0.14 [0.01]	0.21 [0.01]	0.13 [0.01]	0.13 [0.01]	0.17 [0.02]	0.14 [0.01]
Top 10 antigens	***	2.18 [0.12]	2.24 [0.11]	2.07 [0.1]	2.28 [0.1]	2.41 [0.12]	1.89 [0.11]	2.62 [0.09]	1.89 [0.09]	1.97 [0.17]	2.34 [0.17]	1.96 [0.12]
All antigens	***	0.24 [0.03]	0.37 [0.04]	0.24 [0.04]	0.3 [0.04]	0.32 [0.04]	0.23 [0.04]	0.43 [0.04]	0.16 [0.04]	0.21 [0.05]	0.31 [0.06]	0.17 [0.05]
**EAEC**												
Breadth score	***	0.1 [0.01]	0.1 [0.01]	0.07 [0.01]	0.06 [0.01]	0.09 [0.01]	0.06 [0.01]	0.12 [0.01]	0.08 [0.01]	0.11 [0.01]	0.12 [0.02]	0.09 [0.01]
Top 10 antigens	***	1.03 [0.08]	1.03 [0.06]	0.78 [0.08]	0.8 [0.06]	0.9 [0.07]	0.74 [0.06]	1.17 [0.05]	0.9 [0.09]	0.97 [0.09]	1.19 [0.15]	0.86 [0.07]
All antigens	*	0.14 [0.04]	0.19 [0.03]	0.03 [0.04]	0.04 [0.04]	0.09 [0.04]	0.06 [0.04]	0.18 [0.04]	0.07 [0.05]	0.12 [0.05]	0.22 [0.07]	0.07 [0.04]
***Shigella***												
Breadth score	***	0.29 [0.02]	0.29 [0.02]	0.23 [0.02]	0.26 [0.02]	0.27 [0.02]	0.19 [0.02]	0.3 [0.02]	0.19 [0.02]	0.21 [0.02]	0.24 [0.03]	0.19 [0.01]
Top 10 antigens	***	1.89 [0.1]	1.72 [0.09]	1.45 [0.09]	1.62 [0.08]	1.71 [0.1]	1.26 [0.09]	1.74 [0.08]	1.23 [0.07]	1.2 [0.09]	1.35 [0.1]	1.18 [0.07]
All antigens	***	0.71 [0.05]	0.7 [0.05]	0.55 [0.05]	0.63 [0.04]	0.65 [0.05]	0.46 [0.05]	0.7 [0.04]	0.46 [0.04]	0.49 [0.05]	0.6 [0.06]	0.46 [0.04]
***S. enterica* Typhi**												
Breadth score	**	0.16 [0.01]	0.16 [0.01]	0.14 [0.01]	0.14 [0.01]	0.16 [0.01]	0.12 [0.01]	0.17 [0.01]	0.17 [0.01]	0.18 [0.02]	0.21 [0.02]	0.17 [0.01]
Top 10 antigens	***	1.57 [0.09]	1.52 [0.08]	1.22 [0.08]	1.3 [0.08]	1.42 [0.09]	1.12 [0.08]	1.58 [0.07]	1.54 [0.08]	1.55 [0.1]	1.72 [0.13]	1.56 [0.07]
All antigens	**	0.4 [0.03]	0.42 [0.03]	0.34 [0.04]	0.39 [0.03]	0.4 [0.03]	0.29 [0.03]	0.46 [0.03]	0.42 [0.03]	0.46 [0.04]	0.54 [0.05]	0.42 [0.03]
***S. aureus***												
Breadth score	***	0.2 [0.02]	0.16 [0.01]	0.12 [0.02]	0.13 [0.01]	0.15 [0.02]	0.12 [0.02]	0.18 [0.01]	0.25 [0.02]	0.22 [0.02]	0.21 [0.03]	0.23 [0.02]
Top 10 antigens	***	1.65 [0.09]	1.41 [0.08]	1.13 [0.1]	1.29 [0.07]	1.36 [0.1]	1.19 [0.08]	1.54 [0.08]	1.96 [0.1]	1.76 [0.11]	1.71 [0.12]	1.97 [0.09]
All antigens	***	0.46 [0.04]	0.39 [0.04]	0.26 [0.05]	0.31 [0.04]	0.31 [0.05]	0.23 [0.04]	0.39 [0.04]	0.55 [0.07]	0.49 [0.07]	0.47 [0.06]	0.52 [0.05]
***S. pneumoniae***												
Breadth score	***	0.25 [0.01]	0.27 [0.01]	0.2 [0.02]	0.21 [0.01]	0.26 [0.02]	0.22 [0.02]	0.31 [0.02]	0.29 [0.02]	0.28 [0.02]	0.28 [0.03]	0.29 [0.02]
Top 10 antigens	***	2.42 [0.1]	2.24 [0.1]	1.84 [0.1]	1.89 [0.09]	2.16 [0.12]	1.75 [0.1]	2.3 [0.09]	2.33 [0.11]	2.24 [0.13]	2.23 [0.16]	2.42 [0.1]
All antigens	**	0.57 [0.05]	0.66 [0.04]	0.41 [0.05]	0.46 [0.05]	0.6 [0.06]	0.47 [0.05]	0.72 [0.05]	0.61 [0.06]	0.58 [0.07]	0.66 [0.08]	0.64 [0.05]
***M. tuberculosis***												
Breadth score	**	0.24 [0.02]	0.24 [0.02]	0.17 [0.02]	0.18 [0.02]	0.2 [0.02]	0.15 [0.02]	0.23 [0.02]	0.2 [0.02]	0.23 [0.02]	0.23 [0.03]	0.21 [0.02]
Top 10 antigens	***	1.73 [0.1]	1.53 [0.08]	1.18 [0.08]	1.27 [0.08]	1.34 [0.08]	1.12 [0.08]	1.45 [0.07]	1.41 [0.08]	1.53 [0.1]	1.53 [0.11]	1.45 [0.09]
All antigens	**	0.56 [0.05]	0.57 [0.04]	0.35 [0.05]	0.41 [0.04]	0.41 [0.04]	0.33 [0.05]	0.52 [0.04]	0.46 [0.05]	0.52 [0.05]	0.58 [0.07]	0.47 [0.05]

***Effect of cohort via 1-way ANOVA, adj P < 0.001.

**Effect of cohort via 1-way ANOVA, adj P < 0.01.

*Effect of cohort via 1-way ANOVA, adj P < 0.05.

Unexpectedly, IgA reactivity was observed against *M. tuberculosis* proteins in the U.S. and the European Union (E.U.) populations, despite low incidence of tuberculosis in these countries (2016 U.S.: 2.9 cases per 100,000; 2016 Spain 10.7 cases per 100,000; 2015 Sweden <10 cases per 100,000). These data are somewhat perplexing, because the incidence of tuberculosis is much lower in Europe and North America than Africa, with South America having intermediate rates (39, 40). There are several possible explanations for this finding. First, it is possible (but we believe unlikely) that there is a higher incidence of *M. tuberculosis* exposure and/or latent tuberculosis than previously thought in E.U. and U.S. women. It is currently estimated that latent tuberculosis (defined by the absence of clinical symptoms but having positive immunologic responses to the pathogen) affects one third of the global population, and like other members of the genus, *M. tuberculosis* can survive for long periods of time in soil and other media (41). Furthermore, there are reports from the early 1900s that *M. tuberculosis* can be found in the milk of infected women, and breastfeeding has long been known to be a mode of tuberculosis transmission from mother to infant (42, 43). As such, it is possible that these high levels of reactivity indicate higher than anticipated exposure to *M. tuberculosis* in the U.S. and Europe. Speciating the bacterial communities in these milk samples would be needed to test this hypothesis. It is also possible that the antigens chosen to reflect *M. tuberculosis* exposure, although highly responsive to *M. tuberculosis* and clinical signs and symptoms of tuberculosis, are not specific to this species. Supporting this possibility are the findings by Perley and colleagues that showed antibody reactivity against *M. tuberculosis* in non-exposed U.S. individuals overlapping with responses in individuals that had active tuberculosis (44). It is possible that cross-reactivity of milk-borne immunoglobulins to antigens shared by *M. tuberculosis* and other mycobacteria, as have been described for T-cell epitopes, represents important, albeit less specific, protection from these and other related pathogens for the recipient infant (45). It is noteworthy that the high reactivity to *M. tuberculosis*-related antigens in the U.S. and European samples is probably not due to maternal or infant immunization, as tuberculosis vaccination is not currently recommended for broad use in Sweden and Spain, and was never recommended in the U.S. A final, and intriguing possibility is that immunoglobulins to *M. tuberculosis* antigens and to other pathogen antigens observed in healthy women may be cross-reactivity originating from exposure to conserved epitopes in organisms of the gut microbiome, as was described again for T-cell epitopes (46). This hypothesis merits further testing by contrasting pathogen immunoglobulin responses with immunoglobulins against specific antigens in the microbiome.

Compared to IgA, IgG responses had a narrower distribution and they were more often cohort-dependent; *S. aureus* and *S. pneumoniae* IgG were broadly distributed, but *Shigella* spp. and EPEC IgG were mostly detected in Africa and Peru, and *P. falciparum* proteins were limited mainly to African countries (**Figure 1B**, **Table S1B**). For overall IgG reactivity, there was an effect of cohort for all pathogens studied. For instance, IgG reactivity to *Shigella* was higher in Peru than in Spain (*P* < 1x10^-28^, **Table S1B**).

### Distribution of IgA and IgG Breadth Scores Varied by Pathogen and Ig Type

**Figure 2:**
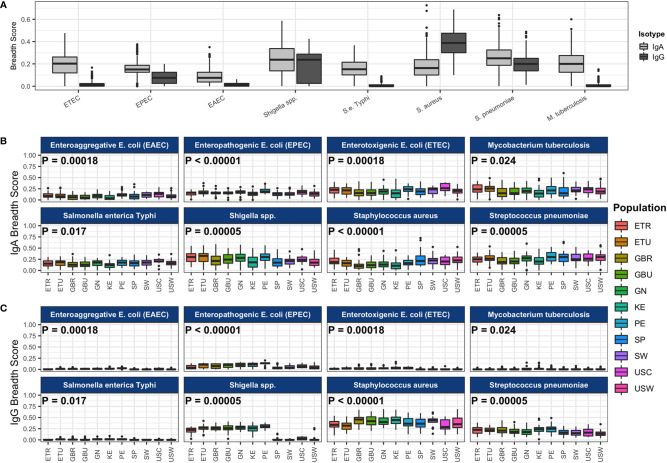
Antibody breadth scores for each pathogen. **(A)** The boxplot represents the distributions of individual subject breadth scores for each pathogen. Breadth score is calculated for each subject for each of the primary pathogens as the proportion of reactive protein array spots among all spots (for each pathogen separately). All study populations are shown together. Immunoglobulin A (IgA) breadth scores are shown in light gray boxes, and immunoglobulin G (IgG) breadth scores are shown in dark gray boxes. Pathogen IgA breadth scores **(B)** and IgG breadth scores **(C)** by population are shown in the colored boxes. P-values from ANOVA of the mean breadth scores among study populations are shown in each pathogen panel. Abbreviations: ETEC, enterotoxigenic *E. coli*; EPEC, enteropathogenic *E. coli*; EAEC, enteroaggregative *E. coli*. Abbreviations: ETR, rural Ethiopia; ETU, urban Ethiopia; GBR, rural The Gambia; GBU, urban The Gambia; GN, Ghana; KE, Kenya; PE, Peru; SP, Spain; SW, Sweden; USC, U.S.-California; USW, U.S.-Washington.

We also calculated antibody breadth scores, a measure of the seropositivity rate (proportion positive) across all antigens for each pathogen. Overall medians and interquartile ranges of IgA and IgG breadth scores for each primary pathogen are illustrated in **Figure 2A**, and IgA and IgG breadth scores for each primary pathogen within each cohort are shown in **Figures 2B, C**. There was an overall effect of cohort on breadth scores for ETEC, EPEC, EAEC, *Shigella*, *S. enterica* Typhi*, S. aureus*, *S. pneumoniae*, *M. tuberculosis*, *P. falciparum*, *O. volvulus*, and measles virus, similar to the observations with IgA and IgG levels, whereby some populations differentially recognized pathogens for IgA or IgG. LMICs had similar antibody breadth scores to HICs, with the exception of at least 5% higher breadth of *Shigella* IgA and IgG (P < 10^-4^ and P < 10^-132^, respectively) and *S. pneumoniae* IgG (P < 10^-10^) and over 5% lower *S. aureus* IgA (P < 10^-10^). IgA recognition of pathogen proteins on the array was widely distributed, with *Shigella* and *S. pneumoniae* each having the highest median breadth scores at approximately 25% of selected proteins recognized (breadth score ≈0.25); maximal IgA recognition was for *S. aureus* at approximately 70% (breadth score ≈0.7, **Figure 2A**). IgG recognition was also high for *Shigella* (25%), *S. aureus* (39%) and *S. pneumoniae* (20%). Importantly, there was a larger selection of proteins for primary pathogens (n≈80) than for secondary pathogens (n=10–15), and possibly greater enrichment for immunodominant proteins in the latter (**Figures 2B, C**). IgA breadth scores were >0 in most samples for all primary pathogens, whereas IgG recognition was near zero in most samples for ETEC, EAEC, *S. enterica* Typhi, and *M. tuberculosis*.

The isotype specificity in human milk likely reflects the route and timing of exposure and invasiveness of bacteria. Specific IgA antibodies and IgA plasma cells in milk and colostrum occur after oral immunization or exposure (47). Such IgA responses are quite dynamic, reflecting the recent exposures in the GI tract (48). However, invasive and systemic infections may efficiently induce IgG responses, which may explain some of the differences in IgA and IgG profiles, although the systemic antibody compartment may also contribute to dimeric IgA profiles in human milk (49). Alternatively, natural, polyreactive IgA and IgM antibodies similar to those that have been described in human milk to self-antigens could explain, to some extent, the broad reactivity of IgA to bacterial pathogens across the cohorts, although they were not reactive to viral proteins (50). The purpose of such natural antibodies in milk is unclear, although they could possibly aid in developing a robust IgA response to non-invasive commensals (51).

### Complex Immunospecificity Patterns Among Cohorts

As described above, significant between-cohort differences in both mean normalized intensity scores and breadth scores were identified by ANOVA for IgA responses to all primary and some secondary study pathogens (**Table 1**, **Figure 1A**, **Figure 2B**, **Table S1A**), and for most pathogen-specific IgG responses (**Table 2**, **Figures 1B** and **2C**, **Table S1B**). However, few patterns emerged from the mean normalized intensity scores except for IgA against EPEC proteins: those from some of the LMIC cohorts tended to be higher than those from Europe and Washington state, U.S.; and European and U.S. cohorts showed a slight tendency for higher IgA levels and breadth against *S. aureus* and *S. pneumoniae*. However, IgG profiles showed stark contrasts, with the most elevated antibody levels in the LMIC cohorts (**Figures 1B** and **2C**), most notably to *Shigella* proteins. The IgG trends agreed with population disease rates, such as shigellosis, which has low incidence in the U.S. and Europe (2013 U.S.: 4.82 cases per 100,000; 2014 Spain: 0.5 cases per 100,000; 2014 Sweden: 3.4 cases per 100,000) (52, 53). Whereas regional variation in IgA levels was evident, the pattern of responses at the continent level for pathogens such as EPEC and *Shigella* showed only slight differences. For primary pathogens, mean normalized intensity scores for milk samples collected in Kenya tended to be the lowest, whereas those in Peru were often the highest.

**Table 2:** Immunoglobulin G (IgG) breadth score and mean [SEM] reactivities to the 10 most-reactive antigens, and mean reactivities to all antigens for each of the primary pathogens included on the protein array in milk produced by women living in rural Ethiopia (ETR), urban Ethiopia (ETU), rural The Gambia (GBR), urban The Gambia (GBU), Ghana (GN), Kenya (KE), Peru (PE), Spain (SP), Sweden (SW), US-California (USC), and US-Washington (USW).

Pathogen	Sig.	ETR	ETU	GBR	GBU	GN	KE	PE	SP	SW	USC	USW
**ETEC**												
Breadth score	**	0.01 [0]	0.02 [0]	0.03 [0]	0.03 [0]	0.03 [0]	0.03 [0.01]	0.03 [0]	0 [0]	0.01 [0]	0.01 [0]	0.01 [0]
Top 10 antigens	**	0.41 [0.03]	0.68 [0.04]	0.72 [0.05]	0.71 [0.04]	0.79 [0.04]	0.75 [0.04]	0.81 [0.04]	0.23 [0.01]	0.25 [0.02]	0.24 [0.02]	0.23 [0.02]
All antigens	**	0.05 [0.01]	0.12 [0.01]	0.13 [0.01]	0.14 [0.01]	0.14 [0.01]	0.15 [0.02]	0.18 [0.01]	0.06 [0.01]	0.07 [0.01]	0.07 [0.02]	0.06 [0.01]
**EPEC**												
Breadth score	**	0.05 [0.01]	0.08 [0.01]	0.07 [0.01]	0.08 [0.01]	0.10 [0.01]	0.1 [0.01]	0.13 [0]	0.04 [0.01]	0.05 [0.01]	0.07 [0.01]	0.05 [0.01]
Top 10 antigens	**	0.98 [0.08]	1.53 [0.13]	1.26 [0.09]	1.4 [0.1]	1.67 [0.12]	1.69 [0.12]	2.37 [0.12]	0.81 [0.07]	0.90 [0.09]	1.34 [0.17]	0.96 [0.11]
All antigens	**	0.08 [0.02]	0.19 [0.02]	0.15 [0.02]	0.18 [0.02]	0.2 [0.02]	0.2 [0.02]	0.34 [0.02]	0.11 [0.01]	0.12 [0.02]	0.18 [0.04]	0.15 [0.02]
**EAEC**												
Breadth score	**	0.01 [0]	0.01 [0]	0.01 [0]	0.01 [0]	0.01 [0]	0.02 [0]	0.03 [0]	0 [0]	0 [0]	0.01 [0]	0 [0]
Top 10 antigens	**	0.25 [0.02]	0.33 [0.02]	0.32 [0.02]	0.33 [0.02]	0.39 [0.02]	0.41 [0.03]	0.58 [0.04]	0.25 [0.02]	0.19 [0.02]	0.36 [0.05]	0.17 [0.01]
All antigens	**	0.03 [0.01]	0.06 [0.01]	0.05 [0.01]	0.06 [0.01]	0.05 [0.01]	0.06 [0.01]	0.11 [0.01]	0.07 [0.01]	0.07 [0.01]	0.08 [0.02]	0.06 [0.01]
***Shigella***												
Breadth score	**	0.21 [0.01]	0.26 [0.01]	0.27 [0.01]	0.26 [0.01]	0.27 [0.01]	0.25 [0.01]	0.3 [0.01]	0.01 [0]	0 [0]	0.03 [0.01]	0.01 [0.01]
Top 10 antigens	**	3.24 [0.21]	4.17 [0.2]	4.16 [0.16]	4.11 [0.2]	4.51 [0.17]	4.24 [0.23]	4.73 [0.13]	0.16 [0.06]	-0.02 [0.01]	0.53 [0.15]	0.18 [0.12]
All antigens	**	0.64 [0.05]	0.95 [0.05]	0.95 [0.05]	0.95 [0.06]	1.04 [0.05]	0.98 [0.07]	1.16 [0.04]	0.02 [0.02]	0 [0.01]	0.08 [0.03]	0.04 [0.03]
***S. enterica* Typhi**												
Breadth score	**	0.01 [0]	0.01 [0]	0.01 [0]	0.01 [0]	0.02 [0]	0.02 [0]	0.01 [0]	0 [0]	0 [0]	0 [0]	0 [0]
Top 10 antigens	**	0.18 [0.02]	0.31 [0.02]	0.41 [0.04]	0.40 [0.04]	0.48 [0.04]	0.38 [0.04]	0.42 [0.03]	0.16 [0.01]	0.15 [0.02]	0.19 [0.04]	0.13 [0.01]
All antigens	**	-0.05 [0.01]	0.01 [0.01]	0.02 [0.01]	0.03 [0.01]	0.04 [0.01]	0.03 [0.01]	0.05 [0.01]	−0.03 [0.01]	−0.03 [0.01]	−0.02 [0.02]	−0.03 [0.01]
***S. aureus***												
Breadth score	**	0.34 [0.02]	0.32 [0.02]	0.43 [0.02]	0.42 [0.02]	0.42 [0.01]	0.44 [0.02]	0.38 [0.02]	0.38 [0.02]	0.42 [0.02]	0.32 [0.04]	0.37 [0.02]
Top 10 antigens	**	3.16 [0.13]	2.80 [0.11]	3.58 [0.11]	3.42 [0.12]	3.53 [0.12]	3.55 [0.14]	3.00 [0.11]	2.75 [0.11]	2.83 [0.13]	2.45 [0.2]	2.72 [0.12]
All antigens	**	1.00 [0.05]	0.89 [0.05]	1.30 [0.06]	1.27 [0.07]	1.25 [0.05]	1.29 [0.07]	1.09 [0.05]	1.06 [0.05]	1.14 [0.07]	0.90 [0.1]	1.03 [0.06]
***S. pneumoniae***												
Breadth score	**	0.21 [0.01]	0.23 [0.01]	0.22 [0.01]	0.20 [0.01]	0.18 [0.01]	0.23 [0.01]	0.24 [0.01]	0.16 [0.01]	0.16 [0.01]	0.17 [0.02]	0.14 [0.01]
Top 10 antigens	**	2.35 [0.11]	2.38 [0.07]	2.59 [0.15]	2.38 [0.13]	2.17 [0.11]	2.60 [0.11]	2.35 [0.09]	1.99 [0.09]	1.94 [0.12]	1.98 [0.18]	1.81 [0.1]
All antigens	**	0.55 [0.04]	0.61 [0.02]	0.63 [0.05]	0.57 [0.04]	0.50 [0.03]	0.66 [0.04]	0.65 [0.03]	0.48 [0.02]	0.48 [0.04]	0.50 [0.06]	0.45 [0.03]
***M. tuberculosis***												
Breadth score	*	0 [0]	0 [0]	0.01 [0]	0.01 [0]	0.01 [0]	0.02 [0.01]	0.01 [0]	0.01 [0]	0.01 [0]	0.01 [0]	0.01 [0]
Top 10 antigens	**	0.26 [0.02]	0.28 [0.02]	0.38 [0.03]	0.38 [0.03]	0.42 [0.03]	0.43 [0.04]	0.37 [0.03]	0.32 [0.02]	0.36 [0.04]	0.35 [0.04]	0.34 [0.03]
All antigens	**	0.05 [0.01]	0.08 [0.01]	0.11 [0.01]	0.13 [0.02]	0.13 [0.02]	0.13 [0.02]	0.13 [0.01]	0.12 [0.01]	0.14 [0.01]	0.1 [0.02]	0.13 [0.01]

**Effect of cohort via 1-way ANOVA, adj P < 0.001.

*Effect of cohort via 1-way ANOVA, adj P < 0.05.

**Figure 3:**
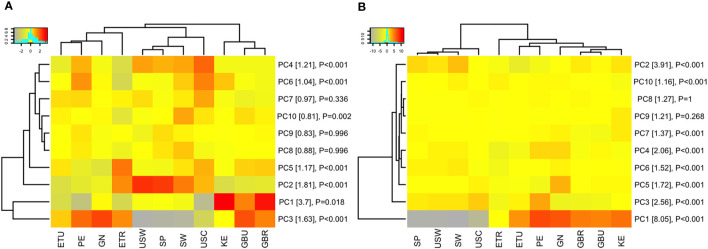
Population clustering by principle component analysis. The heat maps display the mean of each principle component (rows) for each study population (columns). For example, the mean of the first principle component (PC1) for all volunteers in Peru (PE) is summarized in a single value on the heat map. PCA was performed on **(A)** immunoglobulin A (IgA) responses and **(B)** immunoglobulin G (IgG) responses for all *in vitro* transcription and translation (IVTT) proteins represented on the multipathogen protein microarray (purified protein PCA results are shown in the **Supplemental Information**). Only the first 10 PCs are shown, capturing approximately 14% and 25% of the variation in the IgA and IgG data, respectively (a heat map of all PCs are shown in **Supplemental Information**). Populations and PCs were ordered by hierarchical clustering. The color keys show the scale of gray/yellow lower PC values to red higher PC values. Row labels include percent variation captured by each PC in brackets and P-values from ANOVA of the PC means among study populations. Abbreviations: ETR, rural Ethiopia; ETU, urban Ethiopia; GBR, rural The Gambia; GBU, urban The Gambia; GN, Ghana; KE, Kenya; PE, Peru; SP, Spain; SW, Sweden; USC, U.S.-California; USW, U.S.-Washington; PC#, principal component.

Principal component analysis (PCA) was conducted to evaluate whether complex relationships among antigen specificities within pathogen differed across the 11 cohorts. When reducing dimensionality of the data by PCA, antibody profiles clustered by geographic regions (**Figure 3**). Overall, antibody specificity patterns for Sweden, Spain and U.S.-Washington were most similar to each other, with U.S.-California, which specifically recruited mothers who identified as Hispanic, in the same hierarchical clustering branch, but most distant. Patterns for the LMIC cohorts also tended to cluster, although they were split into two separate clusters for IgA responses (**Figure 3A**). The complete set of PCA mean value ANOVA results and PCA loadings are available in the **Supplemental Information** (**Tables S2A, B and S3A, B**). At the pathogen-specific antibody level, there were multiple circumstances where mean values for the LMIC cohorts were categorically different from those of the HIC cohorts; these included, in general, EAEC, EPEC, ETEC, and *Shigella* (**Figures S3A–D**). Notable exceptions included mean values for PC2 and PC1 for complex EAEC- and EPEC-associated antigens, respectively, in rural Ethiopia. Values for rural Ethiopia samples appeared to be different (at least for 2 of the components) from all other cohorts for *M. tuberculosis*, suggesting that complex antigen response by IgA in this cohort was relatively unique. For *S. pneumoniae* and measles, values for Peru tended to be like those for the LMIC cohorts, and for *S. aureus* clustered similarly for Ethiopian populations. The Spain, Sweden, and U.S. cohorts clustered separately from the LMIC cohorts for IgG responses (**Figure 3B**), and clustering of pathogen-specific antigens for IgG followed a similar trend for most pathogens (**Figure S4**). Clustering the data on mean normalized signals, rather than mean PC values, showed similar trends (**Figure S5**).

### Characterization of IgA and IgG Responses to Individual Antigens

Beyond population-level trends in antibody responses to pathogen antigens as a whole, identifying the individual antigens that are recognized by IgA and IgG in human milk may provide an understanding of the immune factors that benefit infants and may protect from disease. **Tables S4A, B** show the reactivity levels to all antigens on the array (ranked by highest reactivity), both globally and specific to each cohort. The highest levels of IgA and IgG antibody binding were observed for specific antigens in each of the six prioritized pathogens associated with diarrheal disease, sepsis, and respiratory illnesses, as follows.

#### *S. aureus*

The strongest IgA response in human milk from all populations was against one of the *S. aureus* exotoxins, also known as superantigen-like protein 7, which is from a family of proteins able to stimulate immune responses regardless of antigen specificity (54). The most reactive IgG response was against the *S. aureus* exotoxin. Additionally, there were strong IgG responses to the components of *S. aureus* leukotoxins gamma-hemolysin, Panton-Valentine leukocidin (PVL), and leukocidin ED (LukED). These are multifunctional, bicomponent leukotoxins that lyse host polymorphonuclear cells such as neutrophils and macrophages (55). IgG was highly reactive to gamma hemolysin components C (hlgC) and B (hlgB), components of PVL LukF-PV and LukS-PV, and the components of LukED, LukE, and LukD; whereas IgA recognition of staphylococcal toxins was primarily to the superantigen exotoxin proteins. This suggests that IgG specificity to staphylococcal proteins is more broadly distributed than IgA responses.

#### EPEC, ETEC, and EAEC

IgA reactivity to EPEC proteins included intimin and the intimin receptor Tir, outer membrane proteins that associate for adherence to epithelial cells, as well as secretory system protein SseC and the secreted protein EspA, which is essential for formation of attaching and effacing lesions during EPEC pathogenesis (56, 57). A membrane protein and a flagellin subunit protein (FliC) were the most IgA reactive proteins of ETEC. For EAEC, an outer membrane autotransporter and conjugal transfer pilus assembly protein (TraB) were most IgA reactive. IgG reactivity was observed against the EPEC proteins Tir, SseC, EspA, and intimin and ETEC putative membrane proteins. EAEC had very low IgG responses.

#### *Shigella* spp

The most IgA reactive for *Shigella* spp. were a *Shigella* hemagglutinin family protein and the invasion plasmid antigen B (IpaB) for *S. flexneri* and *S. sonnei*. The most reactive IgG responses in human milk were also IpaB and other invasion plasmid antigens that overlapped with IgA reactivity.

#### *Salmonella*

The third highest IgA response among all antigens on the array was a *Salmonella* autotransporter protein, an outer membrane-bound translocator protein typically associated with virulence (58). It was also the most IgG-reactive protein for *Salmonella* and showed concordance with IgA reactivity.

#### *S. pneumoniae*

Numerous pneumococcal proteins previously characterized as antibody binding targets ranked among the top reactive for IgA in human milk, including the beta-galactosidase (BgaA), the phage amidase, histidine triad protein E (PhtE), pneumococcal surface protein A (PspA), and the zinc metalloproteases A and B (ZmpA and ZmpB), the latter three being diverse proteins in the pneumococcal population (59). IgG responses to *S. pneumoniae* overlapped with IgA responses, with the addition of the choline binding protein A (PcpA), which was the most reactive pneumococcal protein, is involved in adherence to nasal and lung epithelia, and is a current candidate for subunit vaccines (60). Other *S. pneumoniae* proteins such as LysM and pneumolysin were also among the most reactive antigens (59).

#### *M. tuberculosis*

The most IgA-reactive protein for *M. tuberculosis* was an uncharacterized conserved lipoprotein (LpqN) of unknown function but predicted to be associated with the cell wall and cell processes (61). *M. tuberculosis* had very low IgG responses against all of their proteins

IgA responses to secondary pathogens were heterogeneous, with minimal responses to all the arboviruses, and IgG responses to most secondary pathogen proteins was low. Collectively, the proteins most recognized by IgA in human milk represent diverse functional categories, including toxins and lytic proteins, adhesins, secreted and secretory systems, immune evasion, and antigenic diversity.

Our *a priori* hypotheses were that immunoglobulin immune specificity would vary by pathogen and cohort and be generally higher in LMIC than HIC regions of the world. **Tables S5A, B** show the reactivity levels to all antigens on the array (ranked by ANOVA P-values), both globally and specific to each cohort. **Tables S6A, B** enumerates relative IgA and IgG reactivity to all IVTT antigens on the protein array (as ranked by ANOVA P-value) by cohort for each pathogen. These data, supporting our hypothesis, indicate differences across both pathogens and cohorts in this regard. In total, the ANOVA indicated that at least one of the 11 study cohorts was differentially reactive after adjustment for multiple testing in 357 and 588 of 742 IVTT proteins for IgA and IgG, respectively. A total of 220 and 224 IVTT proteins were identified for IgA and IgG, respectively after filtering for proteins with a positive mean reactivity in at least one cohort (**Table 3**)—positive mean reactivity was defined using mixture models, a method used to empirically define cutoffs for the distribution of positive and negative signals by determining the junction between two Gaussian signal distributions for each protein which has been used for establishing cutoffs for protein microarray summary statistics (**Supplementary Methods, Figures S6A, B**) (62).

**Table 3:** Number (*n*) of *in vitro* transcription and translation (IVTT) proteins on each array for each pathogen; number (*n*) of differentially active proteins across cohorts (*P* < 0.05 *via* 1-way ANOVA) for immunoglobulin A (IgA) and immunoglobulin G (IgG) by pathogen; and number (*n*) of differentially active proteins between lower-to-middle-income countries (LMIC) and high-income countries (HIC) (*P* < 0.05 *via* t-test) for IgA and IgG by pathogen.

Pathogen	IVTT proteins on array	IgA		IgG	
		Proteins with differential activities across cohorts	Proteins with different activities in LMIC vs. HIC	Proteins with differential activities across cohorts	Proteins with different activities in LMIC vs. HIC
EAEC	80	10	2	5	3
EPEC	80	21	14	15	13
ETEC	84	29	13	22	15
*Shigella* spp.	80	39	25	42	36
*S. enterica* Typhi	79	26	14	10	7
*S. aureus*	80	32	23	61	36
*S. pneumoniae*	80	32	17	37	35
*M. tuberculosis*	80	21	7	16	5
*P. falciparum*	15	6	0	13	9
*O. volvulus*	15	2	1	0	0
Measles	12	2	0	2	1
Rubella	10	0	0	1	0
Dengue Virus	12	0	0	0	0
Zika Virus	14	0	0	0	0
Yellow Fever Virus	12	0	0	0	0
Chikungunya Virus	9	0	0	0	0
TOTAL	742	220	116	224	160

**Figure 4:**
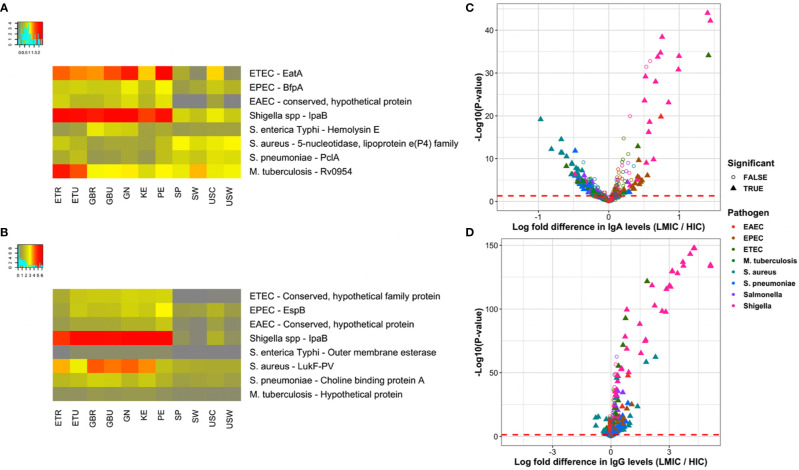
Top differentially reactive antigens per pathogen. **(A, B)** The heat maps present the most significant differentially reactive protein for each of the primary pathogens by ANOVA F-statistic for immunoglobulin A (IgA) and immunoglobulin G (IgG), respectively. ANOVA results for all proteins is included in the **Supplemental Information**. Rows represent per-pathogen proteins with the lowest P-values, and columns represent the mean normalized signals for each study population. All antigens had ANOVA adjusted P-values < 0.05, with the exception of the DENV envelope protein for IgA (P=0.06). **(C, D)** The volcano plots show the log-fold increase in IgA and IgG levels, respectively, between LMIC and HIC cohorts on the x-axis by the inverse log_10_ unadjusted P-value on the y-axis. Each point represents a protein on the multipathogen array that has been colored by pathogen. Filled triangles represent proteins that remain significantly differentially reactive after adjustment, defined as an adjusted P-value < 0.05 and mean normalized signal above the mixed model cutoffs (**Figure S6**) in at least one of the comparator groups. Points to the right of zero indicate higher antibody levels in LMICs, whereas points left of zero indicate higher levels in HICs. Abbreviations: ETEC, enterotoxigenic *E. coli*; EPEC, enteropathogenic *E. coli*; EAEC, enteroaggregative *E. coli*; LMIC, lower- and middle-income country; ETR, rural Ethiopia; ETU, urban Ethiopia; GBR, rural The Gambia; GBU, urban The Gambia; GN, Ghana; KE, Kenya; PE, Peru; SP, Spain; SW, Sweden; USC, U.S.-California; USW, U.S.-Washington.

**Figure 4** depicts averaged normalized signal intensities of relative mean IgA reactivity (**Figure 4A**) and IgG reactivity (**Figure 4B**) to the single most differentially reactive antigen identified for each pathogen. Several patterns emerge from these heatmaps; for instance, reactivity to the top antigen in each of the three *E. coli* strains was generally lower for HICs compared to LMICs. Interestingly, IgA responses to the top antigens of *S. aureus* and *S. pneumoniae* were higher in HICs than in LMICs for IgA, but IgG responses to those were reversed. These patterns held true for many other antigens. In a comparison of LMICs and HICs, 116 and 160 proteins were differentially reactive for IgA (**Figure 4C**) and IgG (**Figure 4D**), respectively (**Table 3**). Among these, IgA against *Shigella* and EPEC proteins were primarily higher in LMICs than HICs, while lower against *S. aureus* and *S. pneumoniae* proteins. Differential reactivity for ETEC and *Salmonella* proteins was heterogeneous—IgA to some proteins were higher in LMICs, while others were higher in HICs. IgG responses were predominantly higher in LMICs, especially for *Shigella* proteins, except for *S. aureus* which had heterogeneous differential reactivity. The list of all antigens in the comparison of IgA and IgG in LMICs and HICs is shown in **Tables S7A, B**, and the top single differentially reactive antigens and clinical relevance are shown in **Table S8**.

The most differentially reactive proteins represented diverse functional categories and had substantial overlap with the most broadly reactive proteins. For example, the most significant *Shigella* IgA and IgG responses were to IpaB and other invasion plasmid antigens, which are multifunctional proteins that are part of the virulence associated genes in the virulence plasmid, involved in the type III secretion system, adhesion, and immune evasion through destruction of phagosomes (63). The *S. flexneri* serine protease A (SepA) is a major extracellular protein involved in disruption of the epithelial barrier during infection and one of the top differentially reactive *Shigella* proteins for both IgA and IgG (64). Likewise, the top ETEC protein (serine protease EatA, a member of an autotransporter family of virulence factors) shares 80% sequence homology with *S. flexneri* SepA and is currently the subject of subunit vaccine development (65). Among other diarrheal pathogen adhesins, IgA against EPEC major structural subunit of bundle-forming pilus BfpA, which is involved in attachment to epithelial cells, and a type II/IV secretion system family protein were highest in LMICs (66). The Peruvian cohort had the highest IgA response to ETEC two-partner secreted adhesin EtpA and EPEC intimin, both involved in epithelial cell adherence (58, 67). ETEC-specific proteins such as EatA and EtpA were positive for IgG in LMICs, but at low normalized intensity. Conversely, ETEC FliC – a major subunit flagellar protein and target for vaccine development – was highest in HICs for IgA and in both HICs and the Peruvian cohort for IgG (68). IgG levels to the EPEC EspB family protein were highest in LMICs. EspB, like *Shigella* Ipa proteins, is a type III secretion system protein and key virulence factor required for attaching and effacing (69). The pneumococcal adhesin PclA, which is a conserved protein found within the *S. pneumoniae* variome had higher IgA reactivity in HICs, but heterogeneous IgG reactivity among the cohorts (70, 71).

Toxins and lytic proteins were another strongly represented class of reactive proteins. *Salmonella* hemolysin E, a pore forming toxin with potential therapeutic antibody application, was reactive in LMICs and mostly nonreactive in HICs; but overall levels were low (**Figure 4A**) (72). ETEC accessory colonization factor YghJ, which has mucinolytic activity and used for intestinal colonization, was particularly high in the Peruvian and U.S.-California cohorts (69). The contrast between IgA and IgG responses to *S. aureus* toxins was observed in multiple exotoxins, as well as the CHAP domain family, which is associated with phage lytic proteins in both bacterial and staphylococcal phage genomes, where IgA reactivity was higher in HICs, while IgG reactivity was higher in LMICs (73). This pattern did not apply to the leukotoxins PVL and LukED. Both IgA and IgG responses to the components of PVL and LukED were higher in LMICs than in HICs. The collinearity of components is expected, since the pore-forming bicomponent leukotoxins must be together to have toxin activity (74). Interestingly, IgA targeting the *M. tuberculosis* ESX-1 secretion-associated protein EspK, a multifunctional protein involved in permeabilization of macrophage phagosomal membranes, was particularly high in rural Ethiopia, but also reactive in all cohorts (75).

Several proteins involved in immune evasion were also differentially reactive. The *S. aureus* sdrE protein is a human factor H-binding protein used for immune evasion of complement, similar to the fHbp protein in *N. meningitidis*, which is a component of a multivalent subunit vaccine (76). Chemotaxis-inhibiting protein CHIPS also evades complement by binding and blocking C5a receptor in neutrophils and monocytes (77). Both had higher IgA reactivity in HICs, although IgG responses were similar between cohorts with the exception of lower IgG reactivity in the Ethiopian cohorts. An EAEC serine protease autotransporter (Pic) is also involved in complement evasion, as well as mucinolytic activity and colonization, and is a protein family secreted by both EAEC and *S. flexneri* (77–79). IgG responses to the highly diverse pneumococcal degradative enzyme immunoglobulin A1 protease, or zinc metalloprotease ZmpA, was lower in HICs (59).

Purified recombinant proteins were printed onto the array for a measure of agreement between two systems of protein expression. The distribution of purified protein signals by population agreed with the trends observed with IVTT proteins (**Figure S7**), except for higher *S. pneumoniae* and *S. aureus* specific IgA levels in LMICs. This deviation may be due to the limited selection of recombinant purified proteins available for printing on the array. Indeed, the individual pneumococcal and staphylococcal purified proteins showing differential reactivity by ANOVA (**Tables S9A, B**) and t-test (**Tables S10A, B**) highlight staphylococcal leukotoxin proteins that were in concordance with higher LMIC IgA against IVTT leukocidins and hemolysins, and the most differentially reactive pneumococcal purified proteins were multiple variants of pneumococcal surface protein A, which had nonsignificant differential reactivity for the IVTT protein and may be influenced by strain specificity (**Table S7A**).

### Biological and Environmental Predictors of Immune Specificity

Linear regression models were used to examine the possibility that various biological and environmental factors were related to variation in immune specificity to the antigens for each pathogen (**Tables S11A, B**). These variables included maternal age, infant sex, time postpartum, parity, delivery mode, maternal BMI, maternal height, presence of companion animals in the home, presence of agricultural animals in the home, and household density. The trends for each factor are shown in volcano plots of the regression coefficients in **Figures S8 and S9**. The most notable associations were with the underweight BMI code; underweight women (BMI < 18.5 kg/m^2^) tended to have higher IgA responses, although none remained significant after adjusting for the false discovery rate. Other factors, notably mother’s age and household density with positive trends and cesarean delivery with a negative trend, showed association with numerous antibodies, but also did not remain significant after adjusting for the false discovery rate.

## Conclusions

Results from this study provide evidence that high-throughput protein array technology can be used to investigate IgA and IgG specificity to a variety of pathogens in human milk underlying substantial infant morbidity and mortality around the world. In addition, our findings support our overarching hypothesis that IgA and IgG specificity varies by pathogen, cohort, and socioeconomic level. Although immune reactivity was generally higher in LMICs than HICs, this was not always true – for instance as it related to IgG reactivity to some antigens associated with *S. aureus*, *S. pneumoniae*, and *M. tuberculosis*. These findings form the basis for additional studies that are needed to determine if variation in milk-borne immunoglobulin reactivity to specific pathogens and/or their antigens is related to variation in infant disease risk and/or severity.

## Data Availability Statement

The original contributions presented in the study are publicly available. This data can be found here: https://www.ncbi.nlm.nih.gov/geo/query/acc.cgi?acc=GSE163903.

## Ethics Statement

The studies involving human participants were reviewed and approved by the Washington State University Institutional Review Board (#13264). The patients/participants provided their written informed consent to participate in this study.

## Author Contributions 

MKM, CM, JW, DS, EK-M, EK, SM, SEM, AP, JF, GO, JR, RP, LB, and MAM designed parent study (the INSPIRE study) and/or oversaw collection of milk samples. MKM, JW, KJ, and AS developed methods for preparation of milk samples for protein array analysis. AR and JC oversaw down-selection of antigens for protein array analysis. KJ and AS provided expertise regarding the immunology of human milk. JC and AR designed and supervised the protein array experiments. AR conducted bioinformatics and statistical analyses. MKM and JC were primary authors for manuscript. All authors contributed to the article and approved the submitted version.

## Funding

Sample collection was funded by the National Science Foundation (award #1344288). Sterile, single-use milk collection kits were kindly provided by Medela, Inc. (McHenry, IL). The Bill and Melinda Gates Foundation (OPP1174492) funded the immunospecificity work which makes up the majority of this report.

## Conflict of Interest

AR and JC are employees of Antigen Discovery Incorporated, a company that carries patents related to the protein array analyses used here and the location where the arrays were conducted.

The remaining authors declare that the research was conducted in the absence of any commercial or financial relationships that could be construed as a potential conflict of interest.

## References

[1] American Academy of Pediatrics Section on Breastfeeding Breastfeeding and the use of human milk. Pediatrics (2012) 129:e827–41. 10.1542/peds.2011-3552 22371471

[2] World Health Organization Exclusive breastfeeding for six months best for babies everywhere. Statement. WHO (2011) Available at: http://www.who.int/mediacentre/news/statements/2011/breastfeeding_20110115/en/, January 15, 2011.

[3] Wold AE Adlerberth I Breast feeding and the intestinal microflora of the infant - implications for protection against infectious diseases. Adv Exp Med Biol (2000) 478:77–93. 10.1007/0-306-46830-1_7 11065062

[4] Verduci E Martelli A Miniello VL Landi M Mariani B Brambilla M Nutrition in the first 1000 days and respiratory health: A descriptive review of the last five years’ literature. Allergol Immunopathol (Madr) (2017) 45(4):405–13. 10.1016/j.aller.2017.01.003 28411961

[5] Oddy WH Infant feeding and obesity risk in the child. Breastfeed Rev (2012) 20(2):7–12. 22946146

[6] Owen CG Whincup PH Cook DG Breast-feeding and cardiovascular risk factors and outcomes in later life: evidence from epidemiological studies. Proc Nutr Soc (2011) 70(4):478–84. 10.1017/S0029665111000590 21801475

[7] Fewtrell MS Breast-feeding and later risk of CVD and obesity: evidence from randomised trials. Proc Nutr Soc (2011) 70(4):472–7. 10.1017/S0029665111000589 21801474

[8] Morozov V Hansman G Hanisch FG Schroten H Kunz C Human milk oligosaccharides as promising antivirals. Mol Nutr Food Res (2018) 62(6):e1700679. 10.1002/mnfr.201700679 29336526

[9] Ackerman DL Craft KM Doster RS Weitkamp JH Aronoff DM Gaddy JA Antimicrobial and antibiofilm activity of human milk oligosaccharides against Streptococcus agalactiae, Staphylococcus aureus, and Acinetobacter baumannii. ACS Infect Dis (2017) 4(3):315–24. 10.1021/acsinfecdis.7b00183 PMC6019282 29198102

[10] Palmeira P Carneiro-Sampaio M Immunology of breast milk. Rev Assoc Med Bras (1992) (2016) 62(6):584–93. 10.1590/1806-9282.62.06.584 27849237

[11] Lönnerdal B Bioactive proteins in human milk: mechanisms of action. J Pediatr (2010) 156(2 Suppl):S26–30. 10.1016/j.jpeds.2009.11.017 20105661

[12] Hunt KM Foster JA Forney LJ Schütte UM Beck DL Abdo Z Characterization of the diversity and temporal stability of bacterial communities in human milk. PloS One (2011) 6(6):e21313. 10.1371/journal.pone.0021313 21695057 PMC3117882

[13] Fernández L Langa S Martín V Maldonado A Jiménez E Martín R The human milk microbiota: origin and potential roles in health and disease. Pharmacol Res (2013) 69(1):1–10. 10.1016/j.phrs.2012.09.001 22974824

[14] Williams JE Carrothers JM Lackey KA Beatty NF York MA Brooker SL Human milk microbial community structure is relatively stable and related to variations in macronutrient and micronutrient intakes in healthy lactating women. J Nutr (2017) 147(9):1739–48. 10.3945/jn.117.248864 PMC5572491 28724659

[15] Williams JE Carrothers JM Lackey KA Beatty NF Brooker SL Peterson HK Strong multivariate relationships exist among milk, oral, and fecal microbiomes in mother-infant dyads during the first six months postpartum. J Nutr (2019) 149(6):902–14. 10.1093/jn/nxy299 PMC6543206 31063198

[16] Lackey KA Williams JE Meehan CL Zachek JA Benda ED Price WJ What’s normal? Microbiomes in human milk and infant feces are related to each other but vary geographically. Front Nutr (2019) 6:45:45. 10.3389/fnut.2019.00045 31058158 PMC6479015

[17] Hassiotou F Hepworth AR Metzger P Tat Lai C Trengove N Hartmann PE Maternal and infant infections stimulate a rapid leukocyte response in breastmilk. Clin Transl Immunol (2013) 2(4):e3. 10.1038/cti.2013.1 PMC4232055 25505951

[18] Pedersen SH Wilkinson AL Andreasen A Kinung’hi SM Urassa M Michael D Longitudinal analysis of mature breastmilk and serum immune composition among mixed HIV-status mothers and their infants. Clin Nutr (2016) 35(4):871–9. 10.1016/j.clnu.2015.05.016 26082337

[19] Hanson LA Breastfeeding provides passive and likely long-lasting active immunity. Ann Allergy Asthma Immunol (1998) 81(6):523–33. 10.1016/S1081-1206(10)62704-4 9892025

[20] Carlsson B Hanson LA Immunologic effects of breast-feeding on the infant. In: Ogra P Lamm ME Strober W McGhee JR Bienestock J, editors. Handbook of Mucosal Immunology. Cambridge, Massachusetts: Academic Press (1994). 10.1016/B978-0-12-524730-6.50057-9

[21] Carbonare SB Silva ML Palmeira P Carneiro-Sampaio MM Human colostrum IgA antibodies reacting to enteropathogenic Escherichia coli antigens and their persistence in the faeces of a breastfed infant. J Diarrhoeal Dis Res (1997) 15(2):53–8. 9360341

[22] Gomes TA Elias WP Scaletsky IC Guth BE Rodrigues JF Piazza RM Diarrheagenic Escherichia coli. Braz J Microbiol (2016) 47:3–30. 10.1016/j.bjm.2016.10.015 27866935 PMC5156508

[23] Walterspiel JN Morrow AL Guerrero ML Ruiz-Palacios GM Pickering LK Secretory anti-Giardia lamblia antibodies in human milk: protective effect against diarrhea. Pediatrics (1994) 93(1):28–31. 8265319

[24] Cleary TG West MS Ruiz-Palacios G Winsor DK Calva JJ Guerrero ML Human milk secretory immunoglobulin A to Shigella virulence plasmid-coded antigens. J Pediatr (1991) 118(1):34–8. 10.1016/S0022-3476(05)81840-2 1986095

[25] Hayani KC Guerrero ML Ruiz-Palacios GM Gomez HF Cleary TG Evidence for long-term memory of the mucosal immune system: milk secretory immunoglobulin A against Shigella lipopolysaccharides. J Clin Microbiol (1991) 29(11):2599–603. 10.1128/JCM.29.11.2599-2603.1991 PMC270380 1774268

[26] Ciardelli L Garofoli F Avanzini MA De Silvestri A Gasparoni A Sabatino G Escherichia coli specific secretory IgA and cytokines in human milk from mothers of different ethnic groups resident in northern Italy. Int J Immunopathol Pharmacol (2007) 20(2):335–40. 10.1177/039463200702000213 17624245

[27] Hayani KC Guerrero ML Morrow AL Gomez HF Winsor DK Ruiz-Palacios GM Concentration of milk secretory immunoglobulin A against Shigella virulence plasmid-associated antigens as a predictor of symptom status in Shigella-infected breast-fed infants. J Pediatr (1992) 121(6):852–6. 10.1016/S0022-3476(05)80327-0 1447644

[28] Crompton PD Kayala MA Traore B Kayentao K Ongoiba A Weiss GE A prospective analysis of the Ab response to Plasmodium falciparum before and after a malaria season by protein microarray. Proc Natl Acad Sci USA (2010) 107:6958–63. 10.1073/pnas.1001323107 PMC2872454 20351286

[29] Cruz-Fisher MI Cheng C Sun G Pal S Teng A Molina DM Identification of immunodominant antigens by hybridization a whole Chlamydia trachomatis open reading frame proteome microarray using sera from immunized mice. Infect Immun (2011) 79(1):246–57. 10.1128/IAI.00626-10 PMC3019893 20956570

[30] Liang L Leng D Burk C Nakajima-Sasaki R Kayala MA Atluri VL Large scale immune profiling of infected humans and goats reveals differential recognition of Brucella melitensis antigens. PloS Negl Trop Dis (2010) 4:e673. 10.1371/journal.pntd.0000673 20454614 PMC2864264

[31] Ruiz L Espinosa-Martos I García-Carral C Manzano S McGuire MK Meehan CL What’s normal? Immune profiling of human milk from healthy women living in different geographical and socioeconomic settings. Front Immunol (2017) 30 8:696. 10.3389/fimmu.2017.00696 PMC5492702 28713365

[32] The World Bank World Bank country and lending groups. The World Bank Group (2020). Available at: https://datahelpdesk.worldbank.org/knowledgebase/articles/906519-world-bank-country-and-lending-groups.

[33] McGuire MK Meehan CL McGuire MA Williams JE Foster J Sellen DW What’s normal? Oligosaccharide concentrations and profiles in milk produced by healthy women vary geographically. Am J Clin Nutr (2017) 105(5):1086–100. 10.3945/ajcn.116.139980 PMC5402033 28356278

[34] Lackey KA Williams JE Price WJ Carrothers JM Brooker SL Shafii B Comparison of commercially-available preservatives for maintaining the integrity of bacterial DNA in human milk. Microbiol Methods (2017) 141:73–81. 10.1016/j.mimet.2017.08.002 28802721

[35] Espinosa-Martos I Montilla A de Segura AG Escuder D Bustos G Pallas C Bacteriological, biochemical, and immunological modifications in human colostrum after Holder pasteurization. J Pediatr Gastroenterol Nutr (2013) 56(5):560–8. 10.1097/MPG.0b013e31828392ed 23274339

[36] Ndungo E Randall A Hazen TH Kania DA Trappl-Kimmons K Liang X A novel Shigella proteome microarray discriminates targets of human antibody reactivity following oral vaccination and experimental challenge. mSphere (2018) 3(4):e00260–18. 10.1128/mSphere.00260-18 PMC6070737 30068560

[37] Davies DH Liang X Hernandez JE Randall A Hirst S Mu Y Profiling the humoral immune response to infection by using proteome microarrays: high-throughput vaccine and diagnostic antigen discovery. Proc Natl Acad Sci USA (2005) 102(3):547–52. 10.1073/pnas.0408782102 PMC545576 15647345

[38] Benjamini Y Hochberg Y Controlling the false discovery rate: a practical and powerful approach to multiple testing. J R Stat Soc B (1995) 57(1):289–300. 10.2307/2346101

[39] Smith I Mycobacterium tuberculosis pathogenesis and molecular determinants of virulence. Clin Microbiol Rev (2003) 16(3):463–96. 10.1128/CMR.16.3.463-496.2003 PMC164219 12857778

[40] World Health Organization Global tuberculosis report. WHO (2017). Available at: http://apps.who.int/iris/bitstream/10665/259366/1/9789241565516-eng.pdf?ua=1.

[41] Ghodbane R Mba Medie F Lepidi H Nappez C Drancourt M Long-term survival of tuberculosis complex mycobacteria in soil. Microbiology (2014) WHO 160(Pt 3):496–501. 10.1099/mic.0.073379-0 24425768

[42] Wang SL Coonley F Occurrence of tubercle bacilli in breast milk of tuberculous women. JAMA (1917) LXIX(7):531–2. 10.1001/jama.1917.02590340031009

[43] World Health Organization Breastfeeding and maternal tuberculosis. WHO (1998). Available at: http://www.who.int/maternal_child_adolescent/documents/pdfs/breastfeeding_and_maternal_tb.pdf?ua=1.

[44] Perley CC Frahm M Click EM Dobos KM Ferrari G Stout JE The human antibody response to the surface of Mycobacterium tuberculosis. PloS One (2014) 9(6):e98938. 10.1371/journal.pone.0098938 24918450 PMC4053328

[45] Lindestam Arlehamn CS Paul S Mele F Huang C Greenbaum JA Vita R Immunological consequences of intragenus conservation of Mycobacterium tuberculosis T-cell epitopes. Proc Natl Acad Sci USA (2015) 112(2):E147–55. 10.1073/pnas.1416537112 PMC4299226 25548174

[46] Scriba TJ Carpenter C Pro SC Sidney J Musvosvi M Rozot V Differential recognition of Mycobacterium tuberculosis-specific epitopes as a function of tuberculosis disease history. Am J Respir Crit Care Med (2017) 196(6):772–81. 10.1164/rccm.201706-1208OC PMC5620682 28759253

[47] Roux ME McWilliams M Phillips-Quagliata JM Weisz-Carrington P Lamm ME Origin of IgA-secreting plasma cells in the mammary gland. J Exp Med (1977) 146:1311–22. 10.1084/jem.146.5.1311 PMC2180976 925605

[48] Hapfelmeier S Lawson MA Slack E Kirundi JK Stoel M Heikenwalder M Reversible microbial colonization of germ-free mice reveals the dynamics of IgA immune responses. Sci (New York NY) (2010) 328(5986):1705–9. 10.1126/science.1188454 PMC3923373 20576892

[49] Fouda GG Eudailey J Kunz EL Amos JD Liebl BE Himes J Systemic administration of an HIV-1 broadly neutralizing dimeric IgA yields mucosal secretory IgA and virus neutralization. Mucosal Immunol (2017) 10(1):228–37. 10.1038/mi.2016.32 PMC5063654 27072605

[50] Vassilev TL Veleva KV Natural polyreactive IgA and IgM autoantibodies in human colostrum. Scand J Immunol (1996) 44:535–9. 10.1046/j.1365-3083.1996.d01-333.x 8947607

[51] Fransen F Zagato E Mazzini E Fosso B Manzari C El Aidy S BALB/c and C57BL/6 mice differ in polyreactive IgA abundance, which impacts the generation of antigen-specific IgA and microbiota diversity. Immunity (2015) 43:527–40. 10.1016/j.immuni.2015.08.011 26362264

[52] Centers for Disease Control and Prevention Foodborne Diseases Active Surveillance Network (FoodNet): FoodNet Surveillance Report for 2011 (Final Report). Atlanta, Georgia: U.S. Department of Health and Human Services, CDC (2012).

[53] European Centre for Disease Prevention and Control Annual Epidemiological Report 2016 - Shigellosis. Stockholm: ECDC (2016). Available at: https://ecdc.europa.eu/en/publications-data/shigellosis-annual-epidemiological-report-2016-2014-data.

[54] Dinges MM Orwin PM Schlievert PM Exotoxins of Staphylococcus aureus. Clin Microbiol Rev (2000) 13(1):16–34. 10.1128/CMR.13.1.16 10627489 PMC88931

[55] Gravet A Colin DA Keller D Girardot R Monteil H Prévost G Characterization of a novel structural member, LukE-LukD, of the bi-component staphylococcal leucotoxins family. FEBS Lett (1998) 436(2):202–8. 10.1016/S0014-5793(98)01130-2 9781679

[56] Hartland EL Batchelor M Delahay RM Hale C Matthews S Dougan G Binding of intimin from enteropathogenic Escherichia coli to Tir and to host cells. Mol Microbiol (1999) 32(1):151–8. 10.1046/j.1365-2958.1999.01338.x 10216868

[57] Kenny B Lai LC Finlay BB Donnenberg MS EspA, a protein secreted by enteropathogenic Escherichia coli, is required to induce signals in epithelial cells. Mol Microbiol (1996) 20(2):313–23. 10.1111/j.1365-2958.1996.tb02619.x 8733230

[58] Benz I Schmidt MA Structures and functions of autotransporter proteins in microbial pathogens. Int J Med Microbiol (2011) 301(6):461–8. 10.1016/j.ijmm.2011.03.003 21616712

[59] Croucher NJ Campo JJ Le TQ Liang X Bentley SD Hanage WP Diverse evolutionary patterns of pneumococcal antigens identified by pangenome-wide immunological screening. Proc Natl Acad Sci USA (2017) 114(3):E357–66. 10.1073/pnas.1613937114 PMC5255586 28053228

[60] Khan MN Sharma SK Filkins LM Pichichero ME PcpA of Streptococcus pneumoniae mediates adherence to nasopharyngeal and lung epithelial cells and elicits functional antibodies in humans. Microbes Infect (2012) 14(12):1102–10. 10.1016/j.micinf.2012.06.007 PMC3490615 22796387

[61] Kapopoulou A Lew JM Cole ST The MycoBrowser portal: a comprehensive and manually annotated resource for mycobacterial genomes. Tuberculosis (Edinb) (2011) 91(1):8–13. 10.1016/j.tube.2010.09.006 20980200

[62] Stone WJR Campo JJ Ouédraogo AL Meerstein-Kessel L Morlais I Da D Unravelling the immune signature of Plasmodium falciparum transmission-reducing immunity. Nat Commun (2018) 9(1):558. 10.1038/s41467-017-02646-2.82 29422648 PMC5805765

[63] Yang SC Hung CF Aljuffali IA Fang JY The roles of the virulence factor IpaB in Shigella spp. in the escape from immune cells and invasion of epithelial cells. Microbiol Res (2015) 181:43–51. 10.1016/j.micres.2015.08.006 26640051

[64] Patel SK Dotson J Allen KP Fleckenstein JM Identification and molecular characterization of EatA, an autotransporter protein of enterotoxigenic Escherichia coli. Infect Immun (2004) 72(3):1786–94. 10.1128/IAI.72.3.1786-1794.2004 PMC356008 14977988

[65] Kumar P Luo Q Vickers TJ Sheikh A Lewis WG Fleckenstein JM EatA, an immunogenic protective antigen of enterotoxigenic Escherichia coli, degrades intestinal mucin. Infect Immun (2014) 82(2):500–8. 10.1128/IAI.01078-13 PMC3911389 24478066

[66] Ramboarina S Fernandes PJ Daniell S Islam S Simpson P Frankel G Structure of the bundle-forming pilus from enteropathogenic Escherichia coli. J Biol Chem (2005) 280(48):40252–60. 10.1074/jbc.M508099200 16172128

[67] Fleckenstein JM Roy K Fischer JF Burkitt M Identification of a two-partner secretion locus of enterotoxigenic Escherichia coli. Infect Immun (2006) 74(4):2245–58. 10.1128/IAI.74.4.2245-2258.2006 PMC1418895 16552055

[68] Roy K Hamilton D Ostmann MM Fleckenstein JM Vaccination with EtpA glycoprotein or flagellin protects against colonization with enterotoxigenic Escherichia coli in a murine model. Vaccine (2009) 27(34):4601–8. 10.1016/j.vaccine.2009.05.076 19523914

[69] Luo Q Kumar P Vickers TJ Sheikh A Lewis WG Rasko DA Enterotoxigenic Escherichia coli secretes a highly conserved mucin-degrading metalloprotease to effectively engage intestinal epithelial cells. Infect Immun (2014) 82(2):509–21. 10.1128/IAI.01106-13 PMC3911403 24478067

[70] Paterson GK Nieminen L Jefferies JM Mitchell TJ PclA, a pneumococcal collagen-like protein with selected strain distribution, contributes to adherence and invasion of host cells. FEMS Microbiol Lett (2008) 285(2):170–6. 10.1111/j.1574-6968.2008.01217.x 18557785

[71] Gámez G Castro A Gómez-Mejia A Gallego M Bedoya A Camargo M The variome of pneumococcal virulence factors and regulators. BMC Genomics (2018) 19(1):10. 10.1186/s12864-017-4376-0 29298677 PMC5753484

[72] Chin CF Lai JY Choong YS Anthony AA Ismail A Lim TS Delineation of B-cell Epitopes of Salmonella enterica serovar Typhi Hemolysin E: Potential antibody therapeutic target. Sci Rep (2017) 7(1):2176. 10.1038/s41598-017-01987-8 28526816 PMC5438399

[73] Zou Y Hou C Systematic analysis of an amidase domain CHAP in 12 Staphylococcus aureus genomes and 44 staphylococcal phage genomes. Comput Biol Chem (2010) 34(4):251–7. 10.1016/j.compbiolchem.2010.07.001 20708437

[74] Meyer F Girardot R Piémont Y Prévost G Colin DA Analysis of the specificity of Panton-Valentine leucocidin and gamma-hemolysin F component binding. Infect Immun (2009) 77(1):266–73. 10.1128/IAI.00402-08 PMC2612235 18838523

[75] Solomonson M Setiaputra D Makepeace KAT Lameignere E Petrotchenko EV Conrady DG Structure of EspB from the ESX-1 type VII secretion system and insights into its export mechanism. Structure (2015) 23(3):571–83. 10.1016/j.str.2015.01.002 25684576

[76] Seib KL Scarselli M Comanducci M Toneatto D Masignani V Neisseria meningitidis factor H-binding protein fHbp: a key virulence factor and vaccine antigen. Expert Rev Vaccines (2015) 14(6):841–59. 10.1586/14760584.2015.1016915 25704037

[77] Postma B Poppelier MJ van Galen JC Prossnitz ER van Strijp JA de Haas CJ Chemotaxis inhibitory protein of Staphylococcus aureus binds specifically to the C5a and formylated peptide receptor. J Immunol (2004) 172(11):6994–7001. 10.4049/jimmunol.172.11.6994 15153520

[78] Abreu AG Fraga TR Granados Martínez AP Kondo MY Juliano MA Juliano L The serine protease Pic From enteroaggregative Escherichia coli mediates immune evasion by the direct cleavage of complement proteins. J Infect Dis (2015) 212(1):106–15. 10.1093/infdis/jiv013 25583166

[79] Harrington SM Sheikh J Henderson IR Ruiz-Perez F Cohen PS Nataro JP The Pic protease of enteroaggregative Escherichia coli promotes intestinal colonization and growth in the presence of mucin. Infect Immun (2009) 77(6):2465–73. 10.1128/IAI.01494-08 PMC2687332 19349428

