# Supplementary material for: Multipathogen Analysis of IgA and IgG Antigen Specificity for Selected Pathogens in Milk Produced by Women From Diverse Geographical Regions: The INSPIRE Study
Source: Front Immunol. 2021 Feb 11;11:614372. doi: 10.3389/fimmu.2020.614372 (PMC7905217; doi:10.3389/fimmu.2020.614372)

Multipathogen analysis of IgA and IgG antigen specificity for selected pathogens in milk produced by women from diverse geographical regions: The INSPIRE Study

Supplemental Information

I. Additional details for Methods
  a. Protein Selection
  b. Statistical Analysis
II. Supplemental Table
III. Supplemental Figures

Methods

Protein selection. The criteria for selection of a protein for inclusion on the multipathogen protein microarray were multileveled. First was selection of pathogens for the array. The primary focus was on gut, respiratory and sepsis-related pathogens that were already available in the Antigen Discovery Inc (ADI) clone library (https://antigendiscovery.com/bacteria). In addition, secondary pathogens were selected to show profiles of antibodies in human milk directed against other diseases of global health concern. Gut-related pathogens selected were enterotoxigenic *E. coli* (ETEC), enteropathogenic *E. coli* (EPEC), enteroaggregatative *E. coli* (EAEC), *Shigella* species (*S. sonnei*, *S. flexneri*, *S. boydii* and *S. dysenteriae*) and *Salmonella enterica* subspecies Typhi. Respiratory-related pathogens were *Mycobacterium tuberculosis* and *Streptococcus pneumoniae*—respiratory syncytial virus (RSV), influenza A, influenza B and *Bordetella pertussis* were not available in the ADI clone library but were available in-house as purified recombinant proteins, which were included on the array. The sepsis-related pathogen was *Staphylococcus aureus* in the clone collection and Group B Streptococcus as purified recominant proteins. Additonal pathogens included measles, rubella, HIV, *Plasmodium falciparum*, *Onchocerca volvulus*, *Haemophilus influenzae* type b (Hib), Dengue virus (DENV), Zika virus (ZIKV), Yellow Fever virus (YFV) and chikungunya virus (CHIKV). Microarray spots were allocated to pathogens to arrive at approximately 900 total pathogen protein spots; approximately 80 spots were assigned for primary pathogens and approximately 15 to secondary pathogens. For each pathogen, summarized data from prior studies performed at ADI were reviewed (see details of prior publications: https://antigendiscovery.com/publications/). Proteins from each pathogen were ranked for selection based on the following criteria: (1) the protein was broadly reactive in patient populations; (2) antibodies correlated with important clinical phenotypes such as protection from disease or infection or correlation with exposure; (3) the protein was identified as immunogenic in the literature though pubmed and google searches of the pathogen name and key words such as “antibody”, “vaccine”, “diagnostic”, “serodiagnostic”, etc. Fulfillment of multiple criteria gave the protein a higher priority ranking. The primary selection criteria for protein inclusion was that antibody reactivity was detected and associated with infection or exposure to the pathogen, such as increased antibody levels following controlled challenge experiments or natural endemic exposure. The secondary criterion for selecting additional proteins after all primary selections were made was that reactivity was detected regardless of differential responses between populations that had been studied, i.e. universally recognized antigens, which allowed inclusion of proteins with strong antibody responses in subjects from both control and infection/disease groups. Thus, there is inherent bias based on the selection process for

broadly reactive proteins, particularly when highlighting responses to top antigens by reactivity (e.g., top 10), resulting in non-differentially reactive antigens being among the targets used to summarize whole pathogen-level responses. This was done to accomplish the primary objective of describing antibody specificities in human milk, whereby reactive but nondifferential antigens are of value. Despite this resulting in greater similarity between different geographical cohorts at the pathogen level, for most pathogens there were individual antigens that had clear differential IgA and/or IgG reactivity across cohorts.

Data processing. The array spots were quantified and saved to an output .gpr file. For each spot on the slide, the .gpr file contains the foreground intensity (median of pixels inside the circle defining the spot) and local background intensity (median of pixels just outside the circle defining the spot). The final raw intensity is the foreground intensity minus the local background intensity. The raw signals were automatically extracted and saved as .csv files in data matrix format, with array spots as rows and samples as columns, using R (http://www.R-project.org).

Statistical analysis. In addition to the methods reported, the data for all pathogen proteins together was analyzed by principal components analysis (PCA) using the base R function "prcomp". PCA values were organized in a matrix with one value per PC per sample. For each cohort, the PC values were summarized as mean per group per PC. Groupwise differences between PC values was analyzed by ANOVA between cohorts and were visualized by heat maps using the "heatmap.2" function in the "gplots" R package. Both PCs and cohort groups were allowed to cluster using the built-in heirarchical clustering option. To complement the PCA analysis, groupwise mean normalized signal intensities for each pathogen protein were rendered as heat maps with heirarchical clustering. In addition, the data for each pathogen was analyzed by PCA separately to show pathogen-specific profiles as described above. To classify antigens as reactive or non-reactive to IgA or IgG at the population-level, mixture models were applied using the "mixtools" R package to empirically fit Guassian distributions of negative and positive global means of each pathogen protein. Global means were calculated by taking the mean of all study samples for each antigen on the multipathogen array. The mean and three standard deviations of the negative population was used to define a cutoff for positivity, which was used to identify antibody reactivity at the population level, i.e. mean normalized signal over cutoff in one cohort is classified as a reactive antigen for that cohort. Human milk IgA and IgG responses were fit separately. Importantly, this procedure provides an unbiased cutoff point for determination of antibody reactivity. This procedure was a post hoc analysis based on procedures employed in concurrent published work with proteome microarrays [61]. All descriptive statistics and groupwise statistical tests (i.e. T-tests and ANOVA) between study cohorts, PCA analysis and multivariable linear regression were *a priori* planned analyses. Grouping of cohorts into LMIC and HIC populations and differential analysis was *post hoc* based on need to further reduce data dimensionality.

Supplemental Table

Supplemental Table 8

**Top Pathogen Differentially Reactive Antigen**	**Clinical Relevance**	**References**
IgA-reactive		
ETEC - EatA	vaccine candidate, virulence factor	65
EPEC - BfpA	adhesion, secretion	66
EAEC – Conserved, hypothetical protein	unknown	-
Shigella spp. - IpaB	virulence factor, secretion, adhesion, immune evasion	63
S. enterica Typhi – Hemolysin E	antibody therapeutic candidate, toxin	72
S. aureus – 5-nucleotidase lipoprotein e(P4) family	unknown	-
S. pneumoniae - PclA	adhesion, invasion, diversity	70
M. tuberculosis – Rv0954	unknown	-
IgG-reactive		
ETEC – Conserved, hypothetical protein	unknown	-
EPEC – EspB	virulence factor, secretion	69
Shigella spp. - IpaB	virulence factor, secretion, adhesion, immune evasion	63
S. enterica Typhi – Outer membrane esterase	unknown	-
S. aureus – LukF-PV	leukotoxin	55
S. pneumoniae – PcpA	vaccine candidate, adhesion	60
M. tuberculosis – Hypothetical protein	unknown	-

Supplemental Figures

Figure S1

A

ETEC IgG, # of clones with signal		
	SC112516	JL112015
>10000	6	27
>20000	1	6
>30000	1	1

ETEC IgA, # of clones with signal		
	SC112516	JL112015
>10000	1	17
>20000	1	7
>30000	0	5

B

SA IgG, # of clones with signal		
	SC112516	JL112015
>10000	54	54
>20000	26	22
>30000	14	13

SA IgA, # of clones with signal		
	SC112516	JL112015
>10000	4	34
>20000	2	11
>30000	1	2

C

SE IgG, # of clones with signal		
	SC112516	JL112015
>10000	4	2
>20000	0	2
>30000	0	1

SE IgA, # of clones with signal		
	SC112516	JL112015
>10000	6	20
>20000	2	6
>30000	1	6

D

Sample	Enterococcus faecalis	Salmonella typhimurium	Lactobacillus reuteri	Escherichia coli	Streptococcus equi	Enterobacter cloacae	Morganella morganii
JL	0.209	1.080	0.340	0.238	> 1	0.061	0.178
SC	0.015	0.470	0.025	0.024	0.027	0.028	0.018

E

SC112516	IgA w/o BSA	IgA w/ BSA	sIgA w/o BSA	sIgA w/ BSA
IgA w/o BSA	1	0.98	0.97	0.98
IgA w/BSA	0.98	1	0.97	0.98
sIgA w/o BSA	0.97	0.97	1	0.99
sIgA w/BSA	0.98	0.98	0.99	1

JL112015	IgA w/o BSA	IgA w/ BSA	sIgA w/o BSA	sIgA w/ BSA
IgA w/o BSA	1	0.95	0.90	0.93
IgA w/BSA	0.95	1	0.80	0.88
sIgA w/o BSA	0.90	0.80	1	0.96
sIgA w/BSA	0.93	0.88	0.96	1

Figure S2

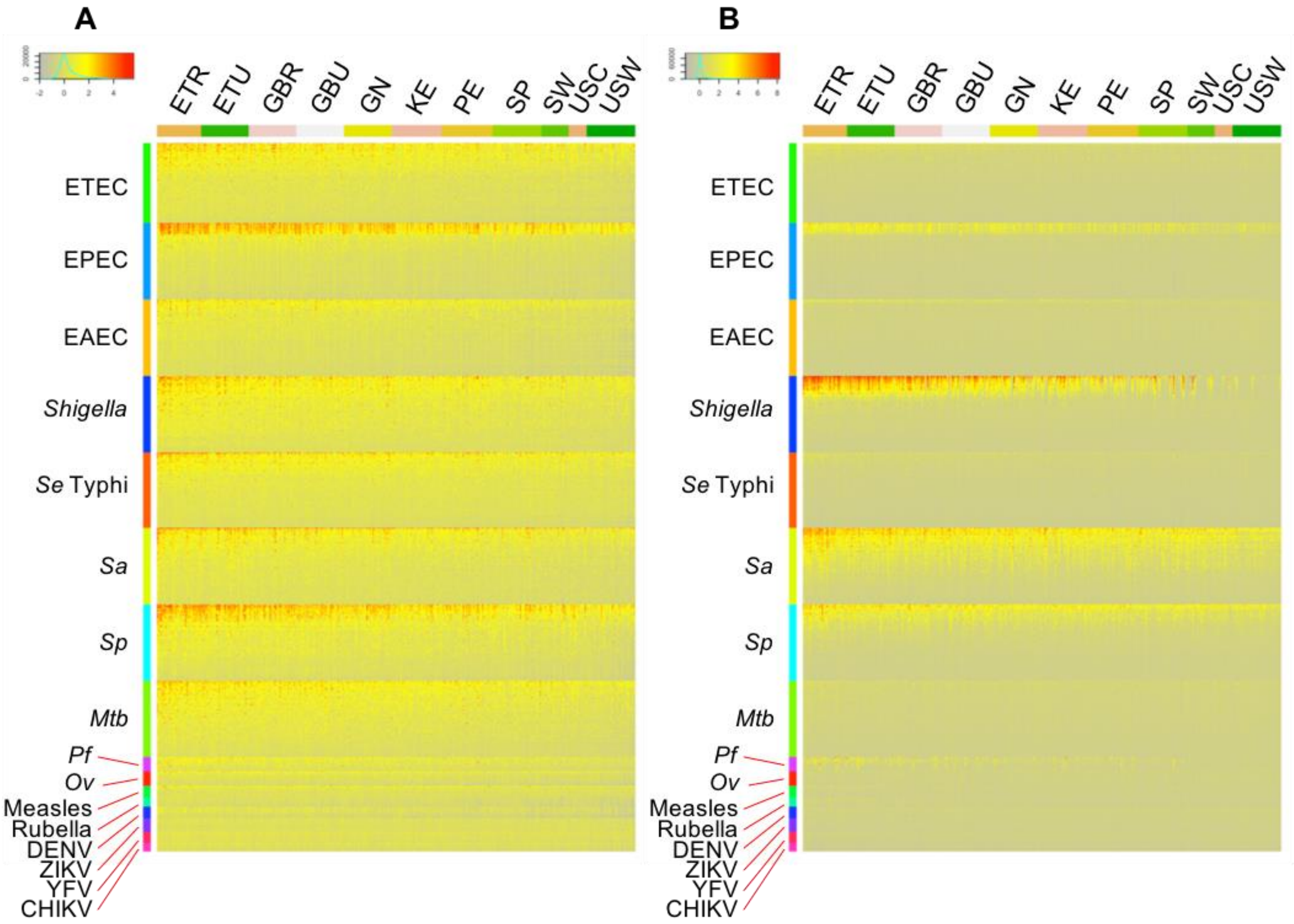
A
B
ETR
ETU
GBR
GBU
GN
KE
PE
SP
SW
USC
USW
ETEC
EPEC
EAEC
Shigella
Se Typhi
Sa
Sp
Mtb
Pf
Ov
Measles
Rubella
DENV
ZIKV
YFV
CHIKV

Figure S3. Population clustering by PCA, Pathogen-specific IgA

A. EAEC

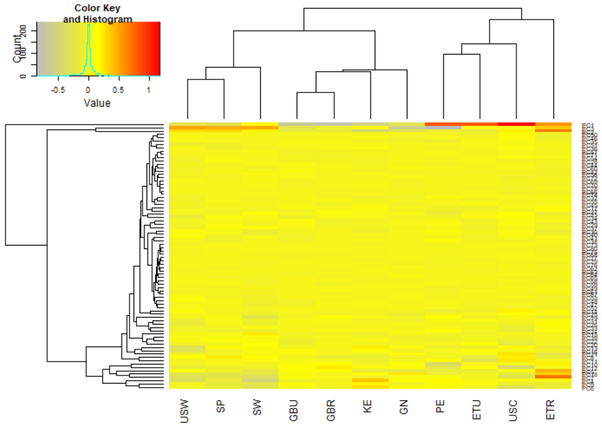

B. EPEC

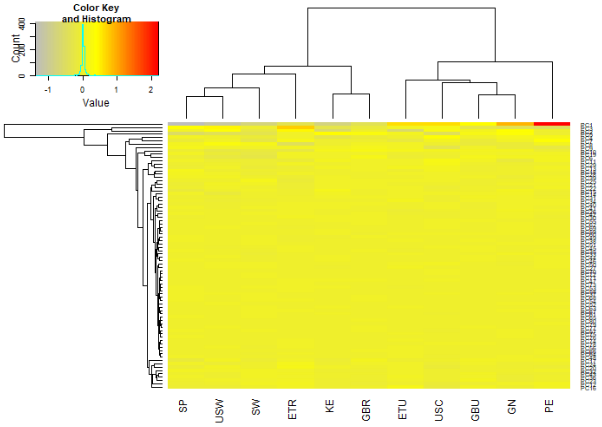

C. ETEC

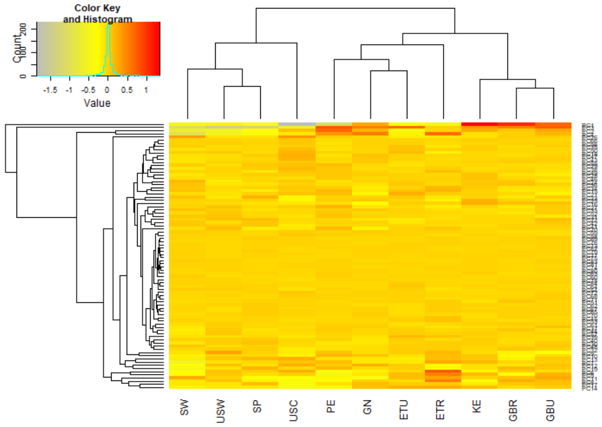

D. *Shigella*

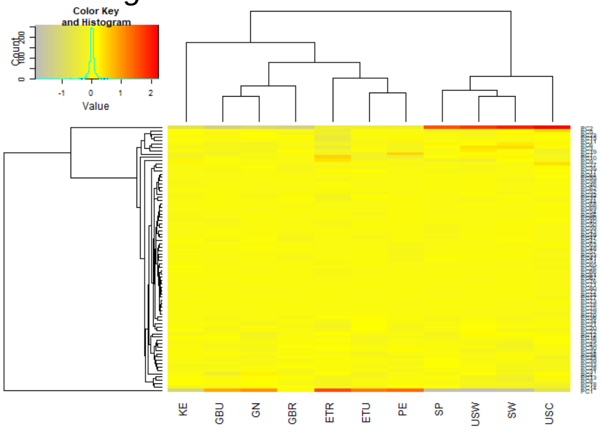

E. *Salmonella enterica* Typhi

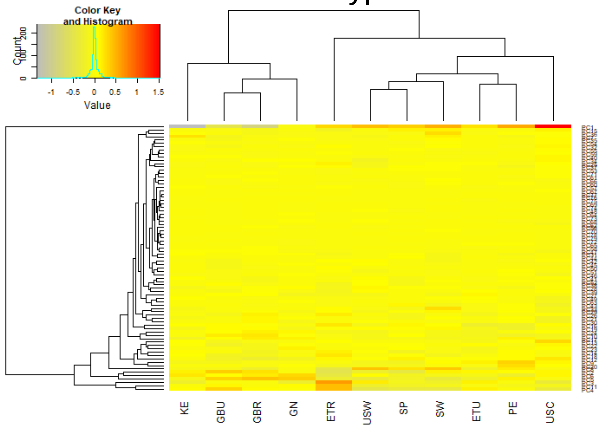

F. *Mycobacterium tuberculosis*

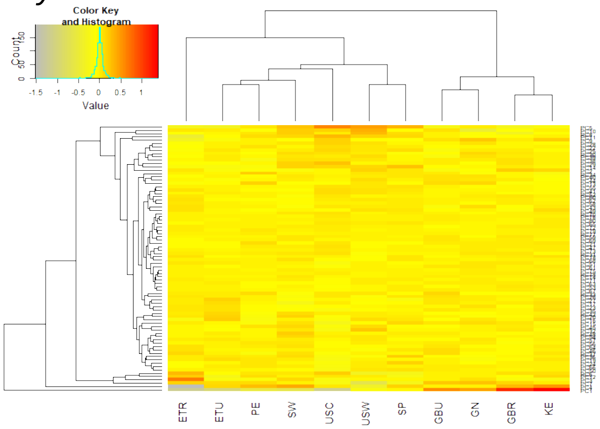

G. *Streptococcus pneumoniae*

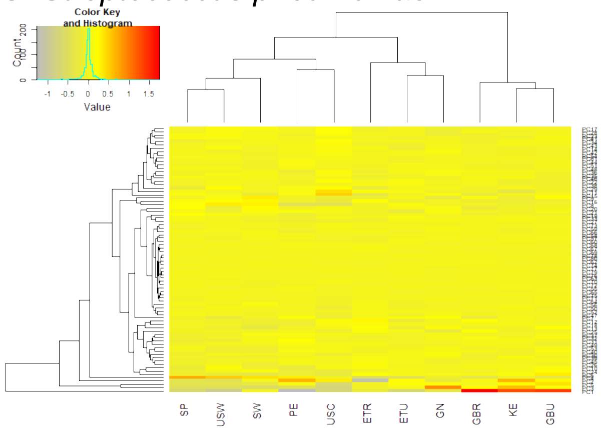

H. *Staphylococcus aureus*

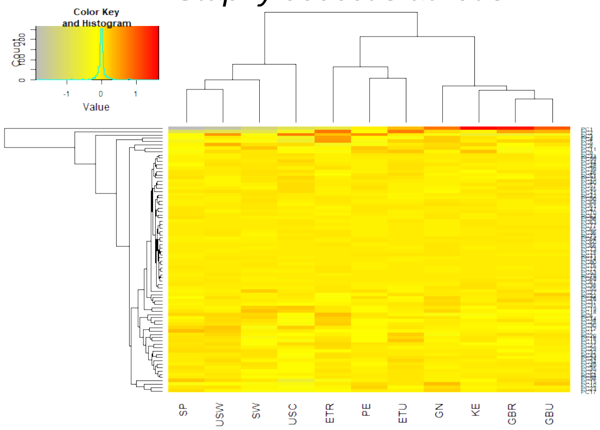

I. *Plasmodium falciparum*

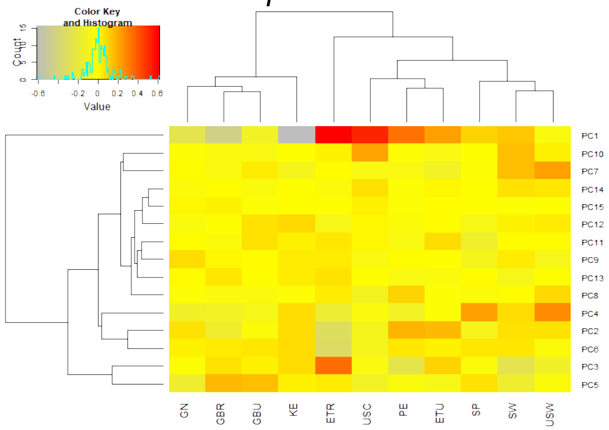

J. *Onchocerca volvulus*

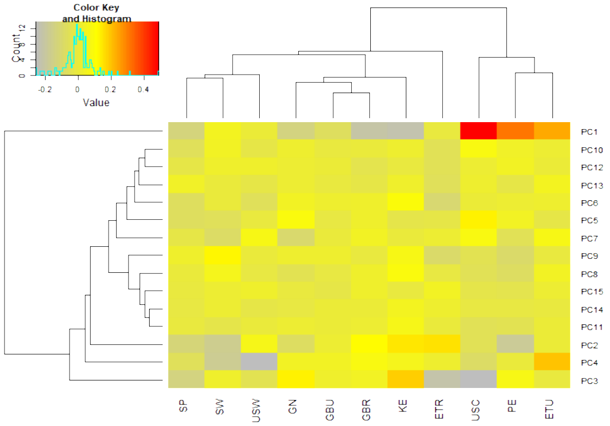

K. Measles

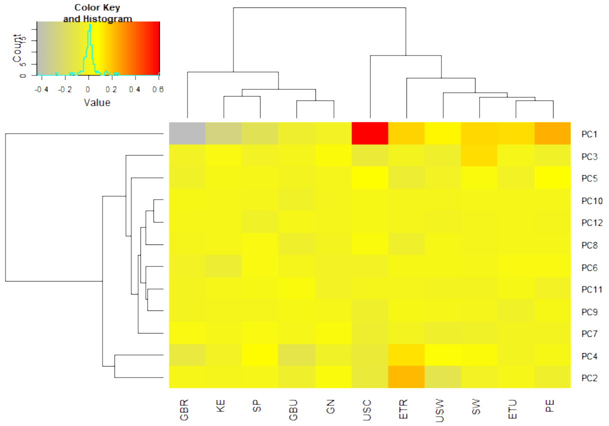

L. Rubella

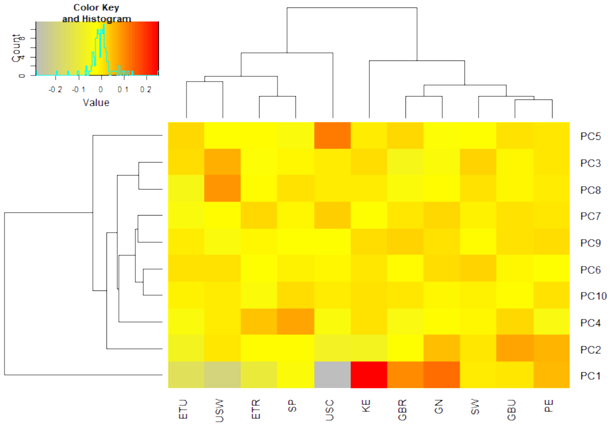

M. Dengue Virus

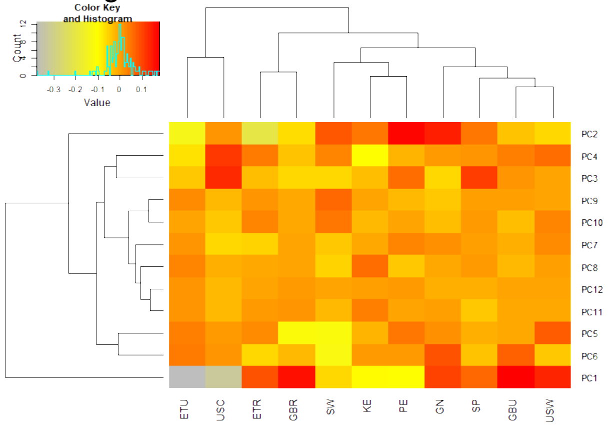

N. Zika Virus

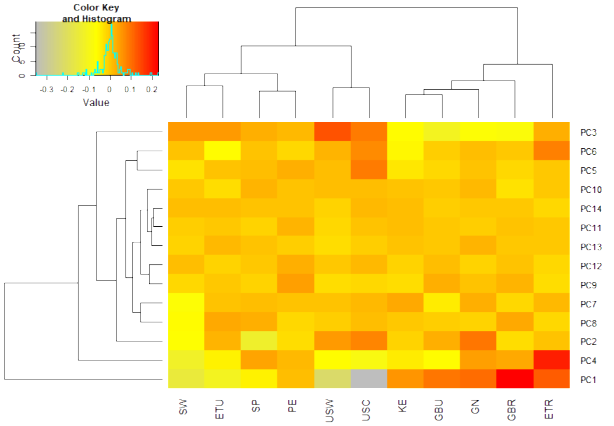

O. Yellow Fever Virus

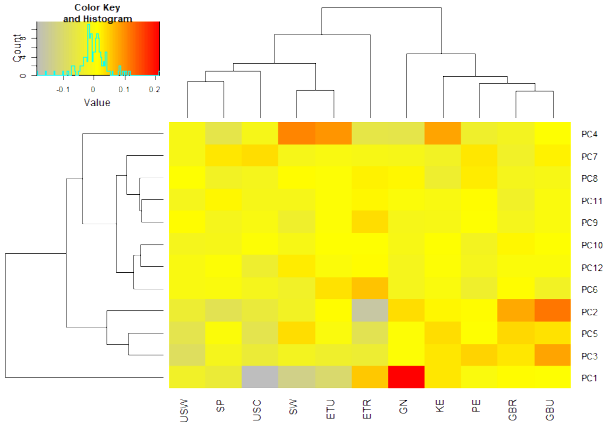

P. Chikungunya Virus

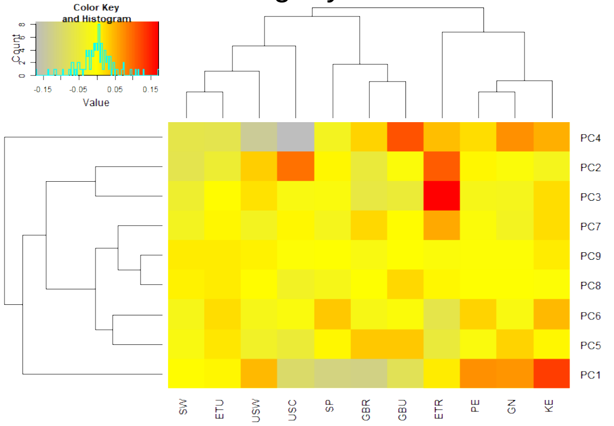

Figure S4. Population clustering by PCA, Pathogen-specific IgG

A. EAEC

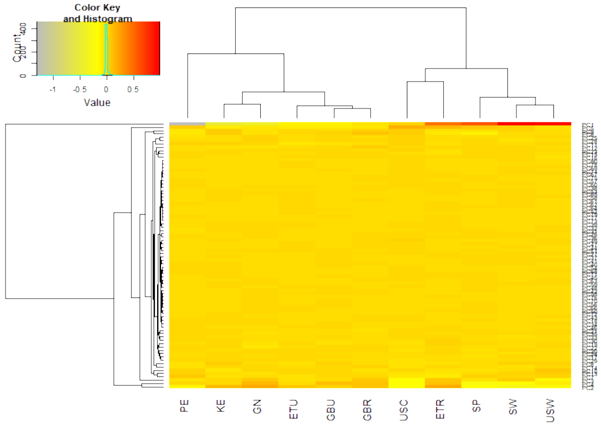

B. EPEC

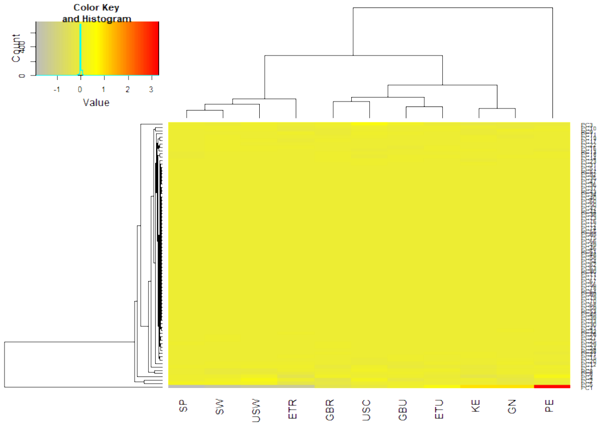

C. ETEC

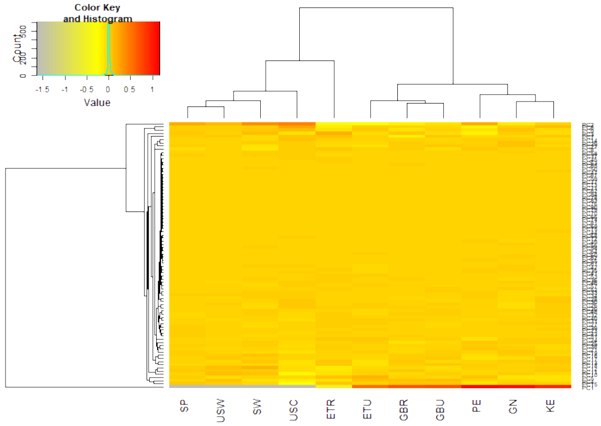

D. *Shigella*

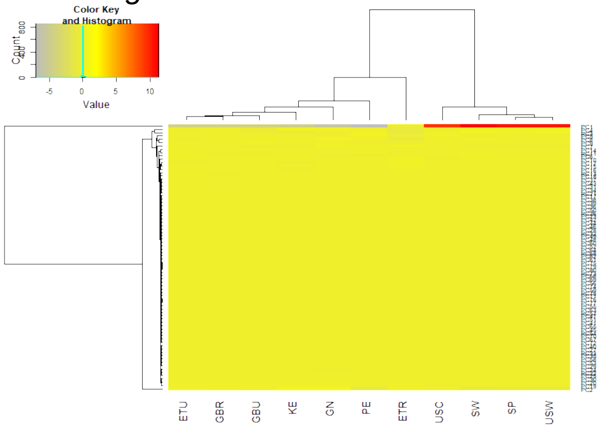

E. *Salmonella enterica* Typhi

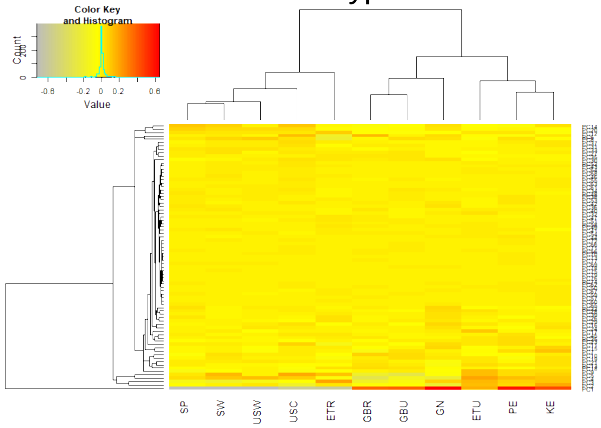

F. *Mycobacterium tuberculosis*

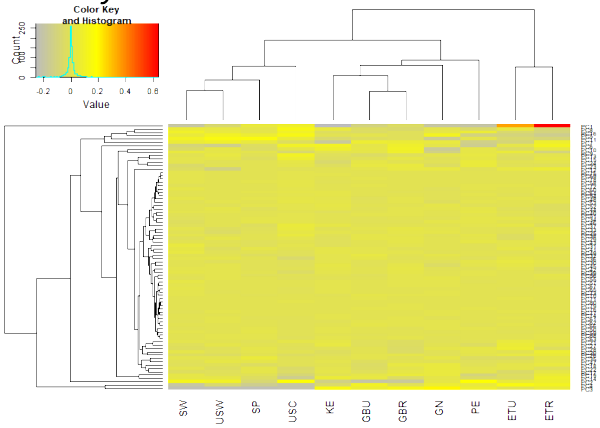

G. *Streptococcus pneumoniae*

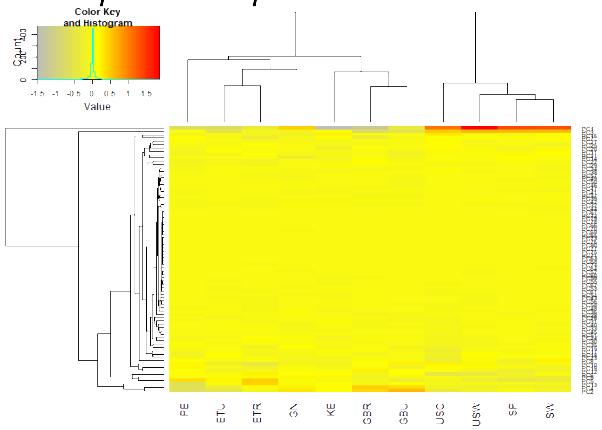

H. *Staphylococcus aureus*

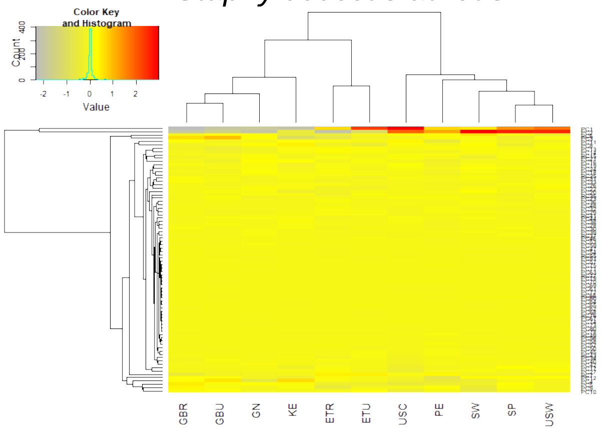

I. *Plasmodium falciparum*

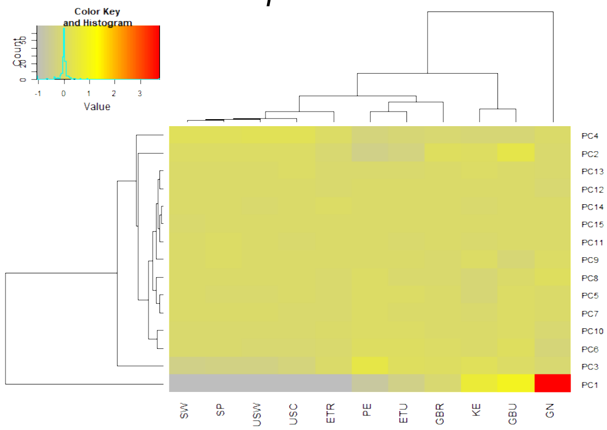

J. *Onchocerca volvulus*

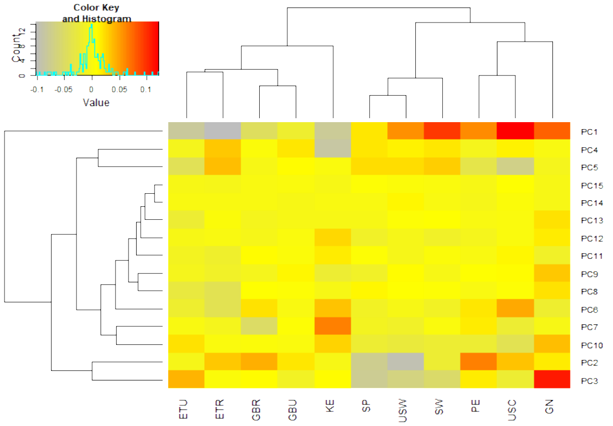

K. Measles

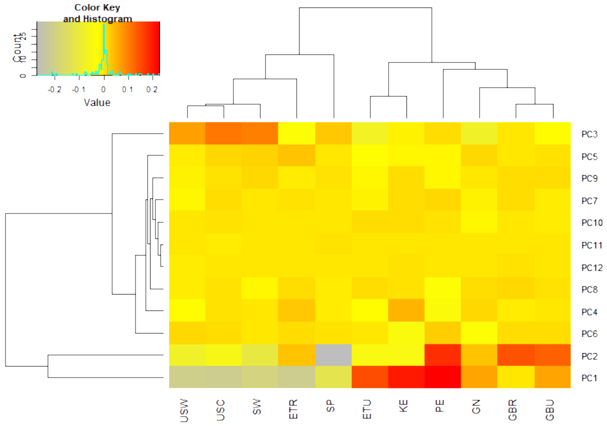

L. Rubella

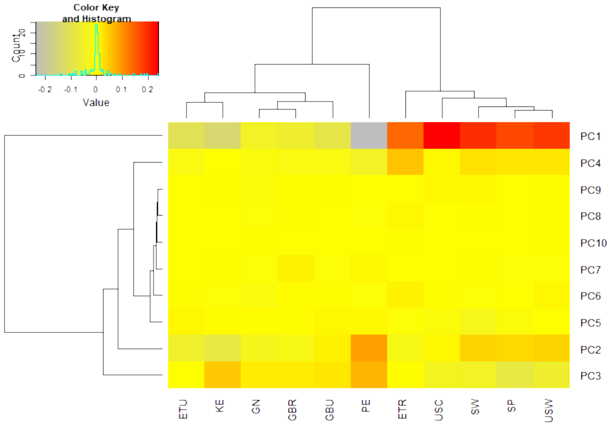

M. Dengue Virus

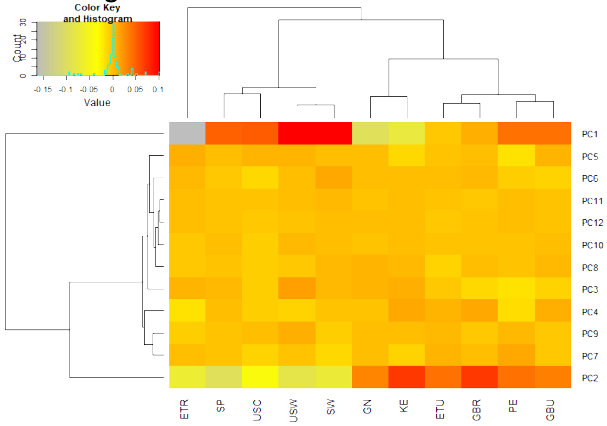

N. Zika Virus

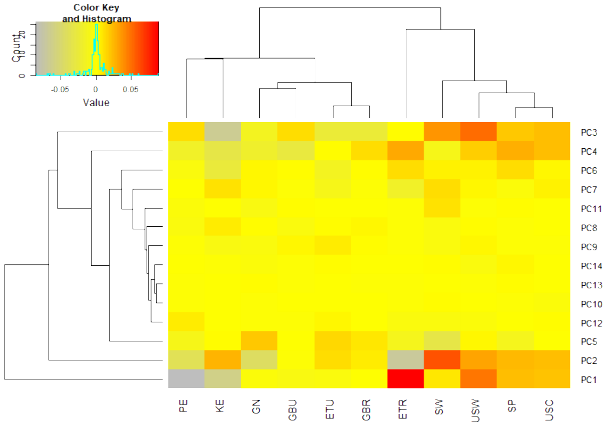

O. Yellow Fever Virus

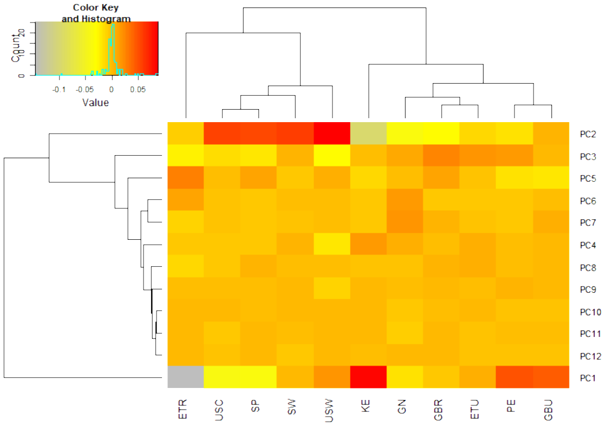

P. Chikungunya Virus

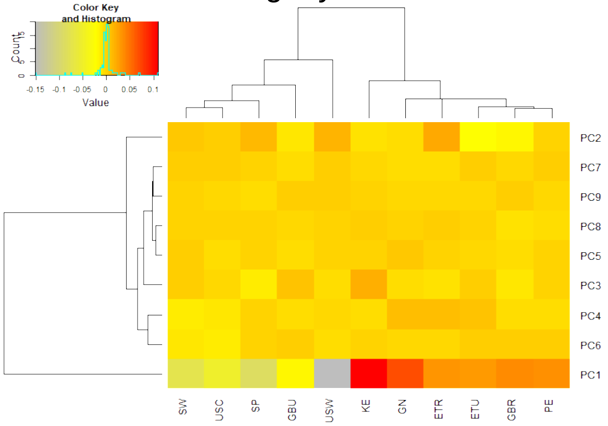

Figure S5. Population clustering by group means for all antigens

A. IgA group means

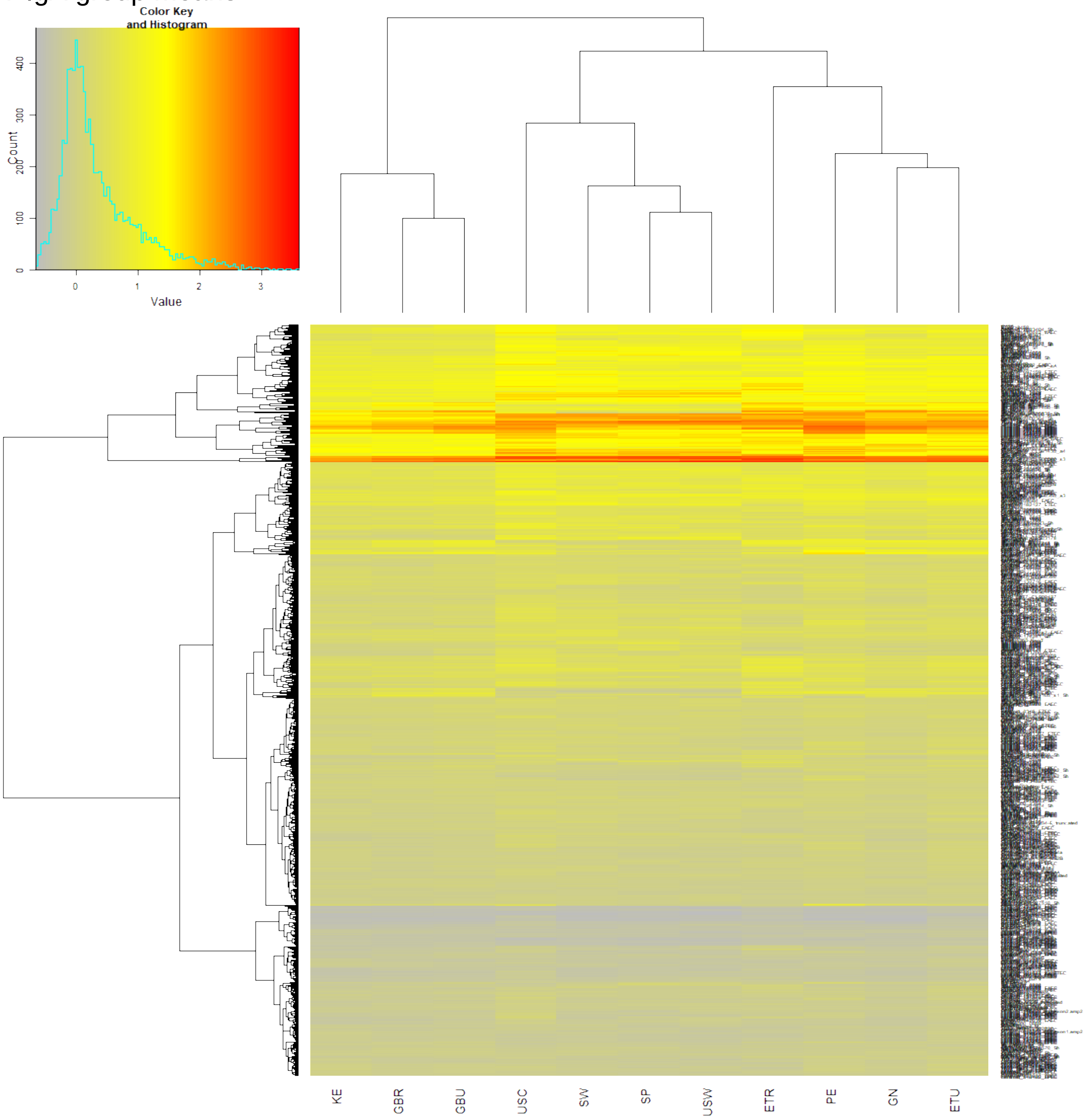

B. IgG group means

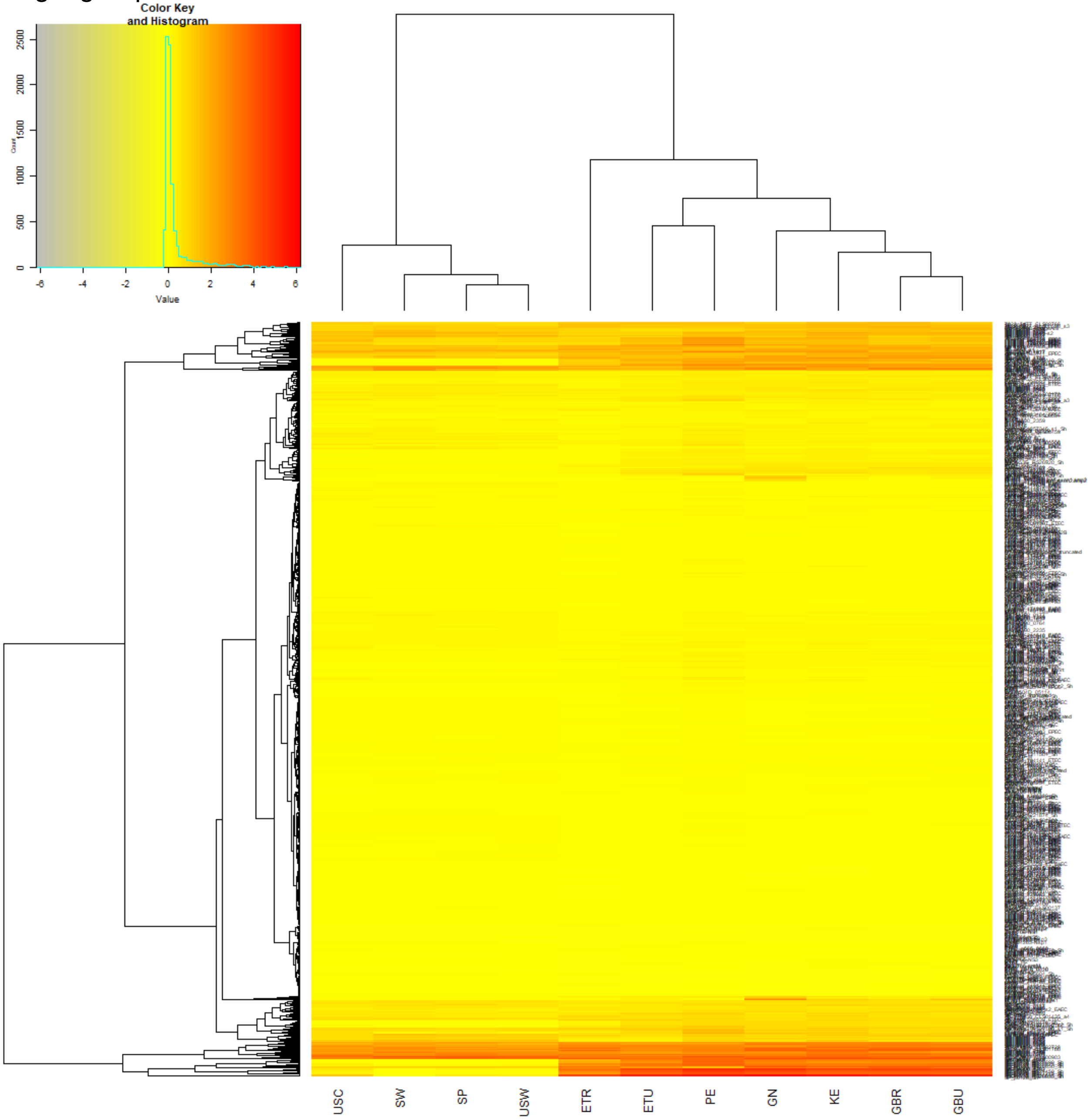

Figure S6. Mixture models

A.

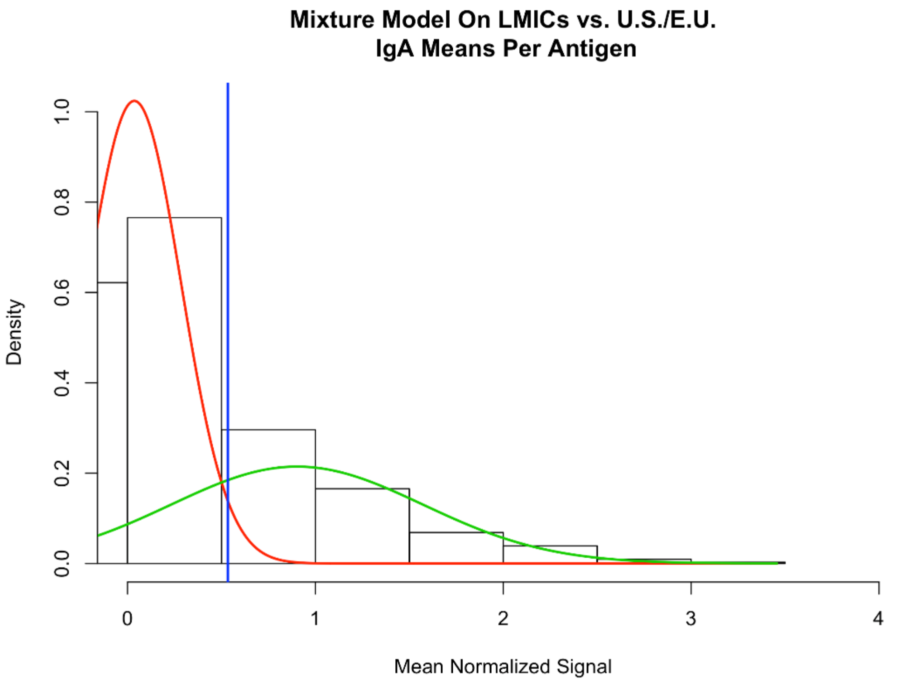

B.

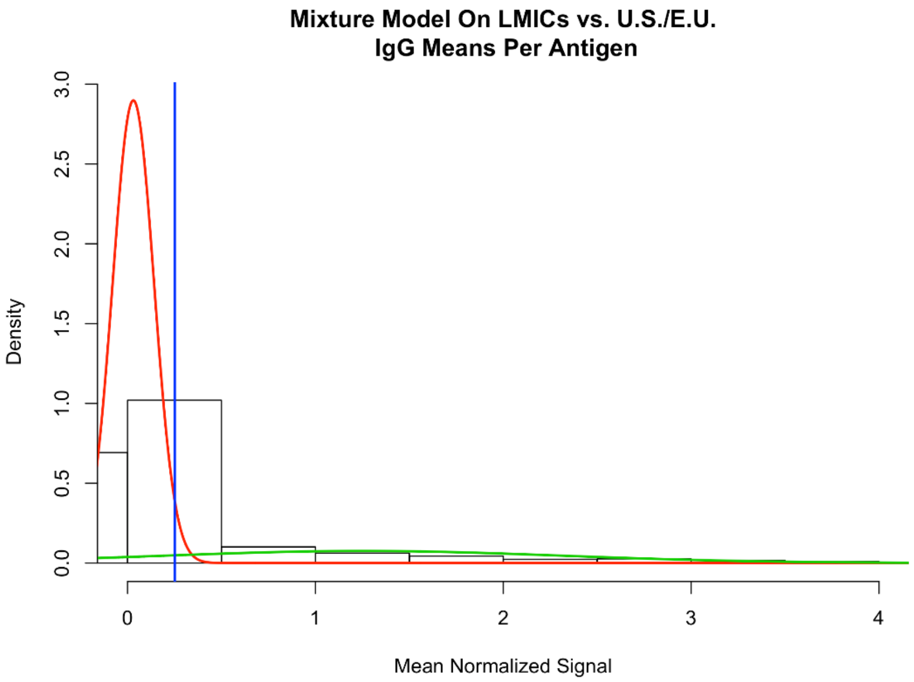

Figure S7. Population-level IgA and IgG reactivity to specific pathogen proteins (purified proteins)

A. IgA responses

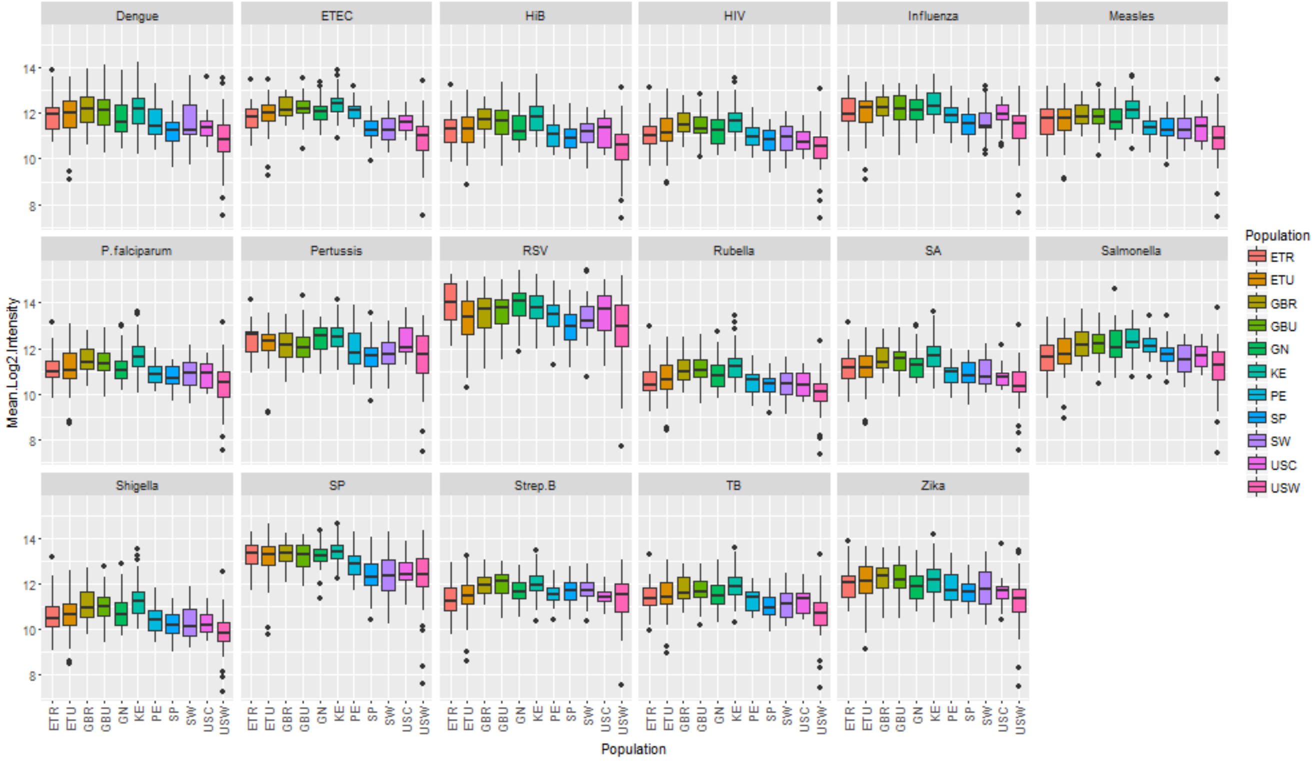

B. IgG responses

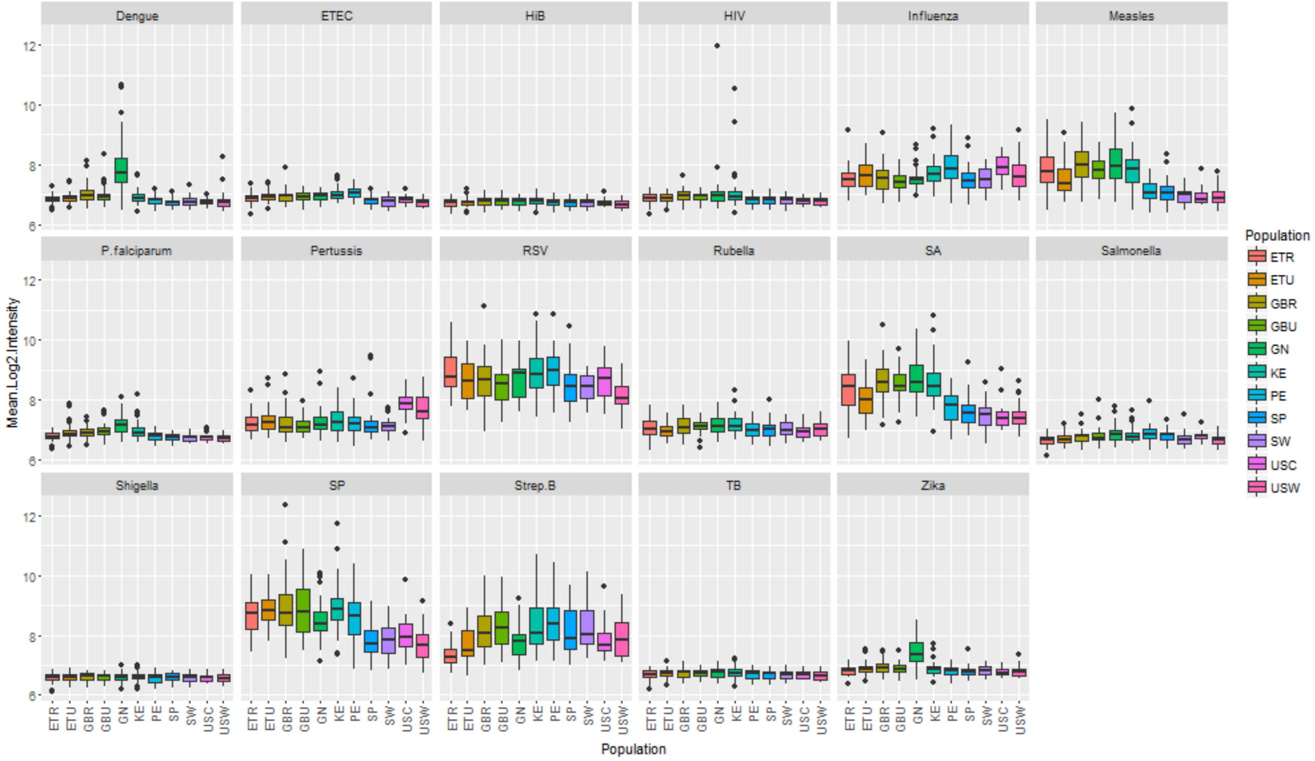

Figure S8. Biological and environmental factors associated with IgA specificity

A. Mother’s age

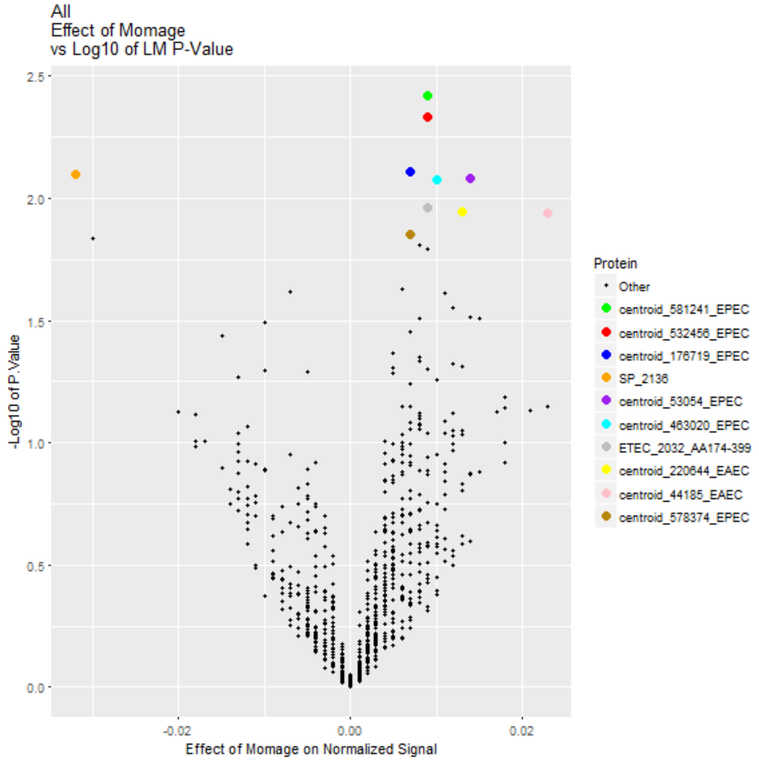

B. Infant sex

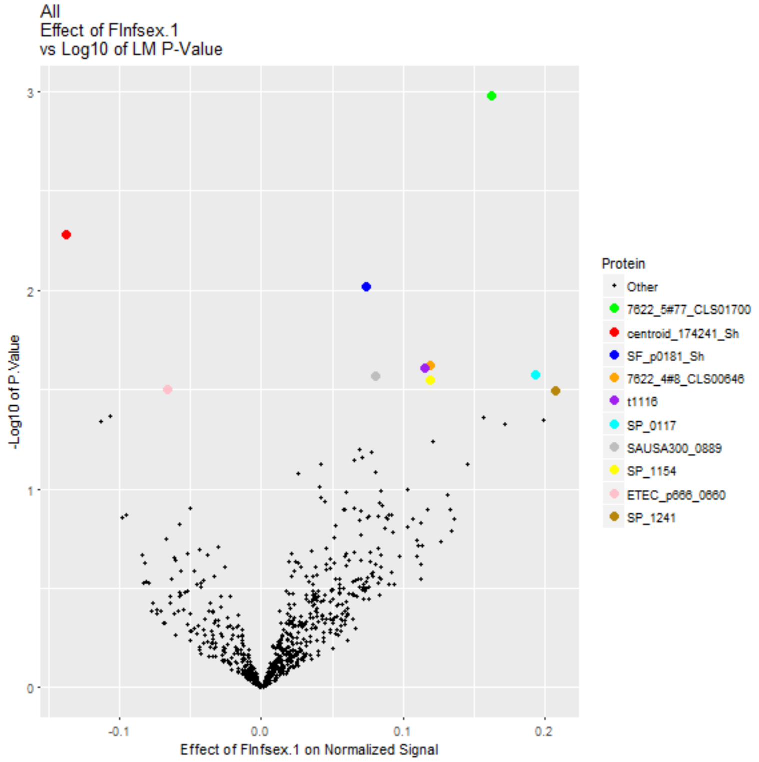

C. Time post-partum

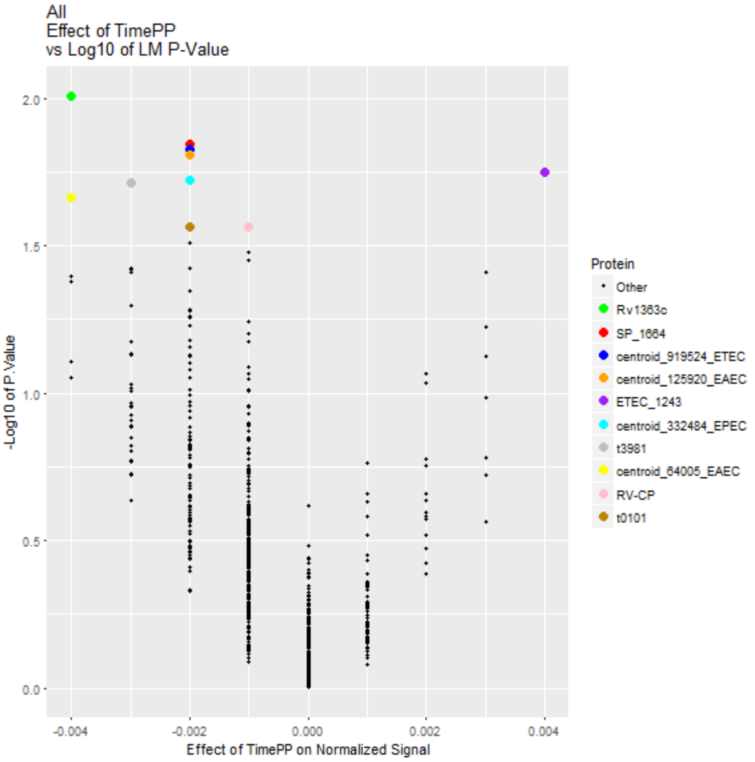

D. Parity

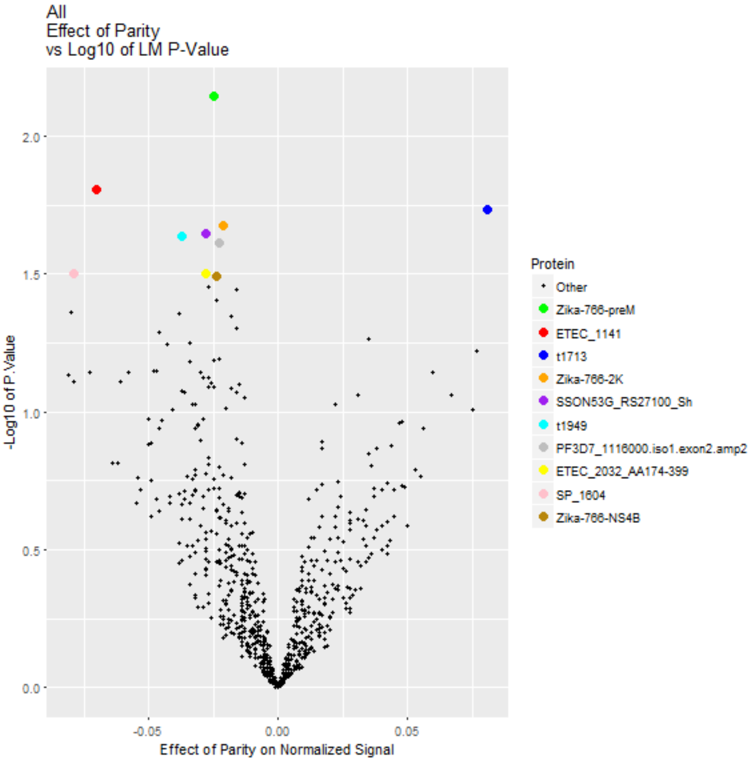

E. Cesarean delivery

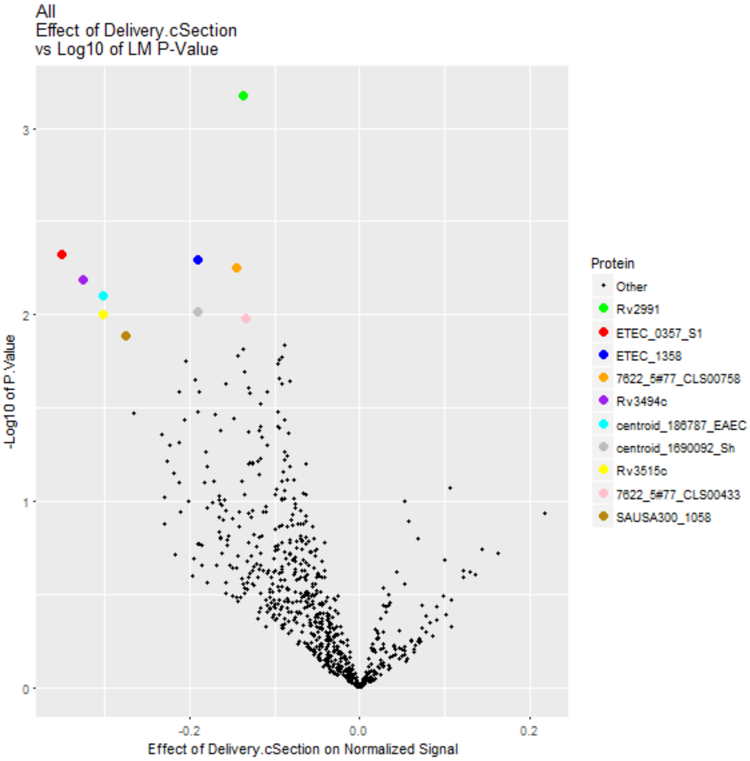

F. BMI code: underweight

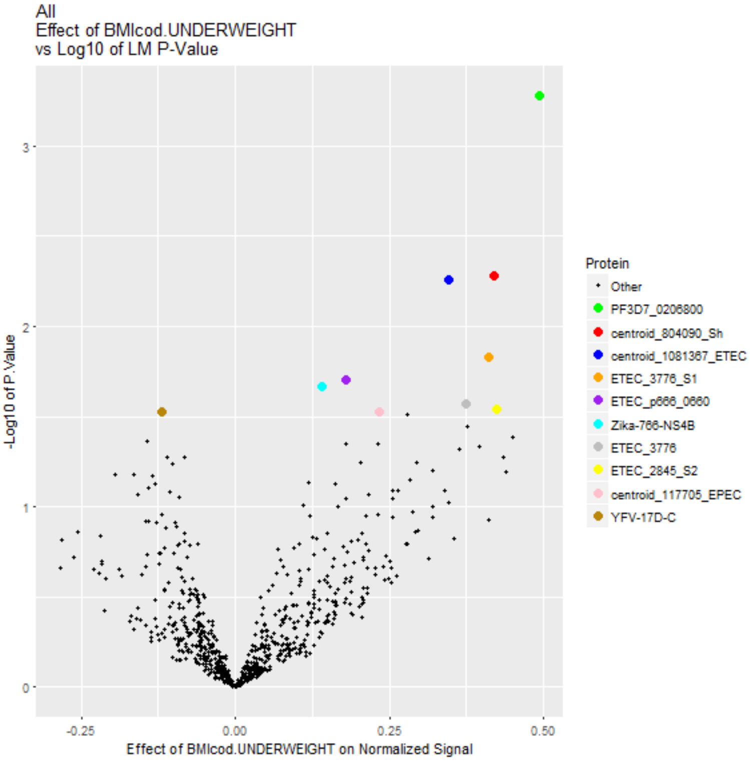

G. BMI code: overweight

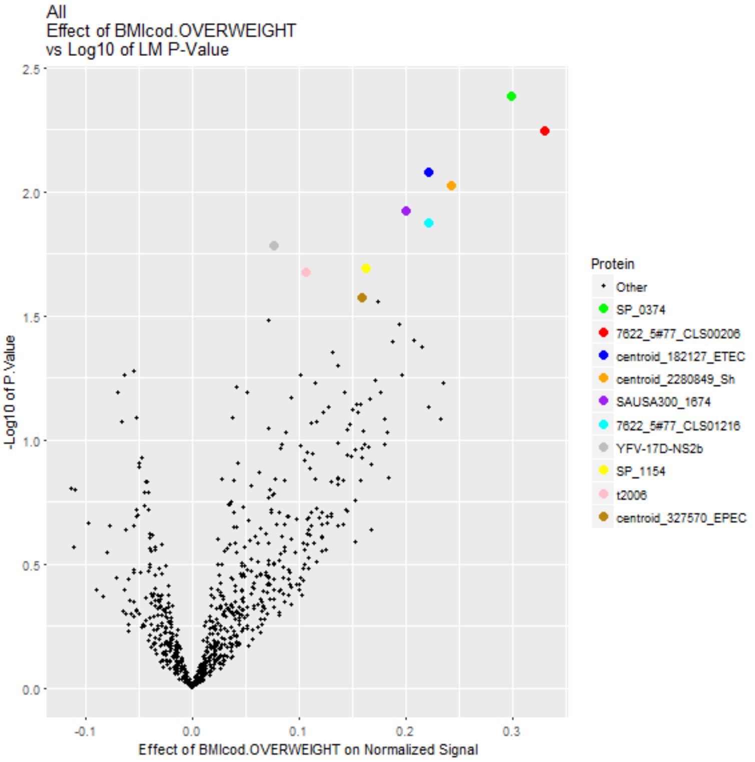

H. BMI code: obese

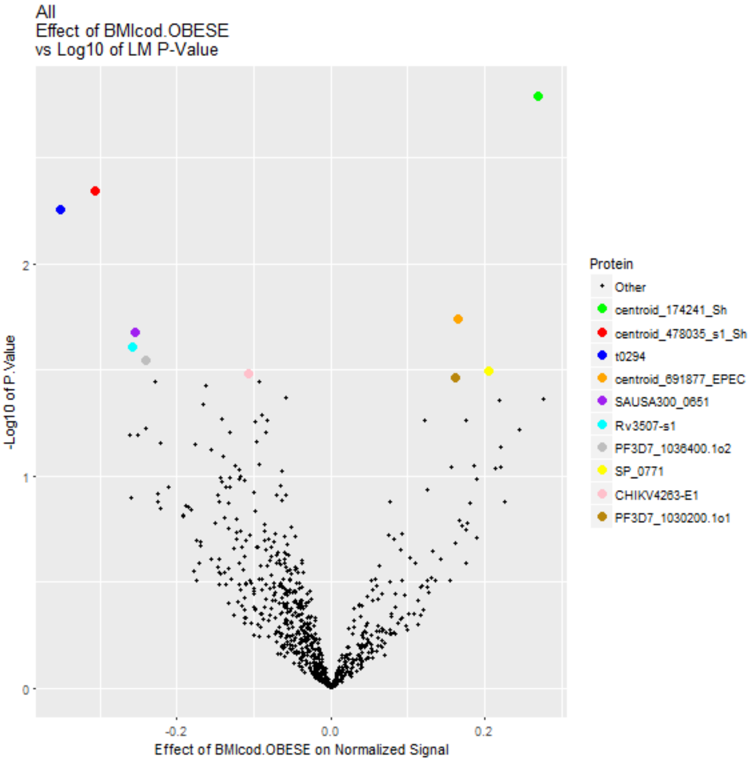

I. Mother's height

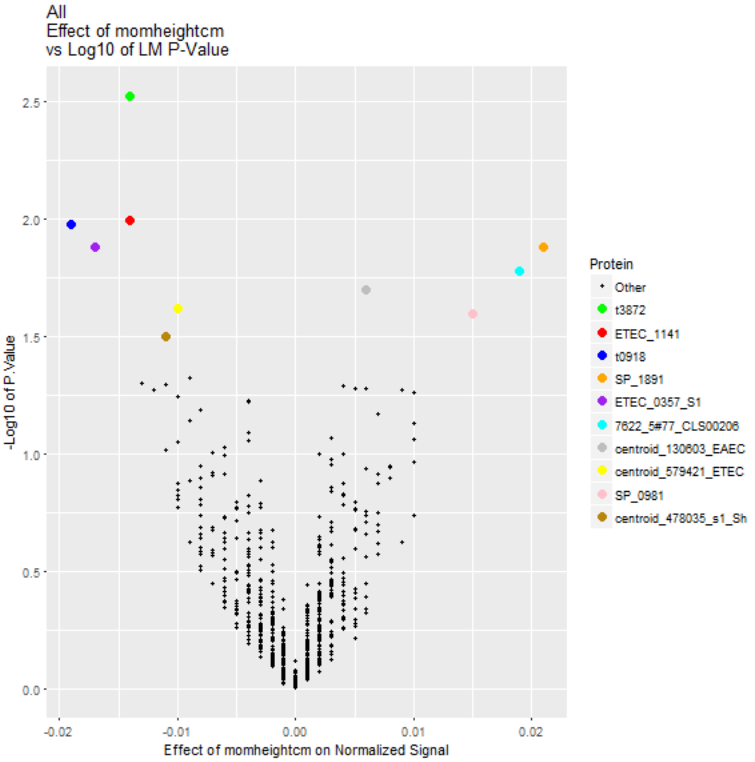

J. Companion animals in household

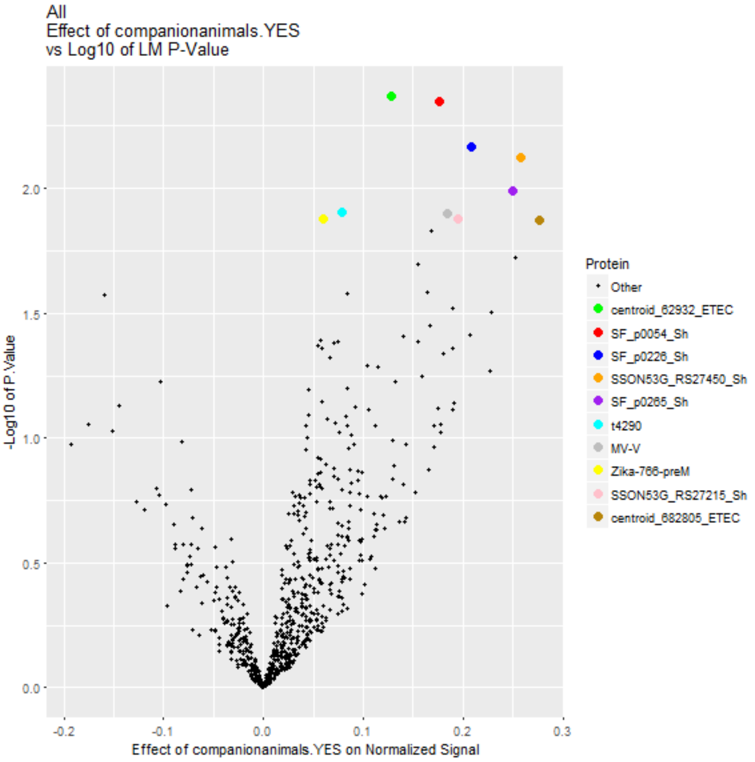

K. Agricultural animals

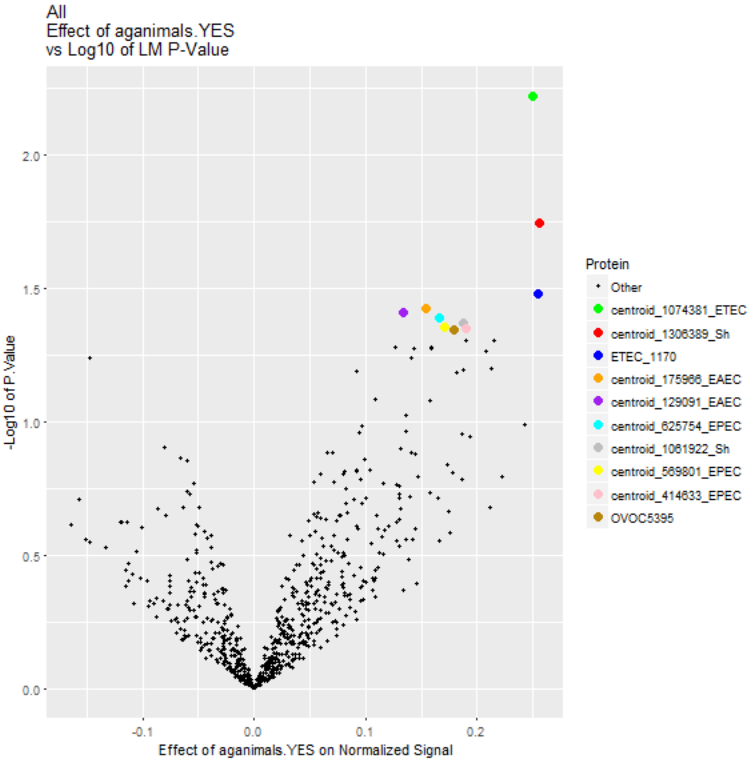

L. Household density

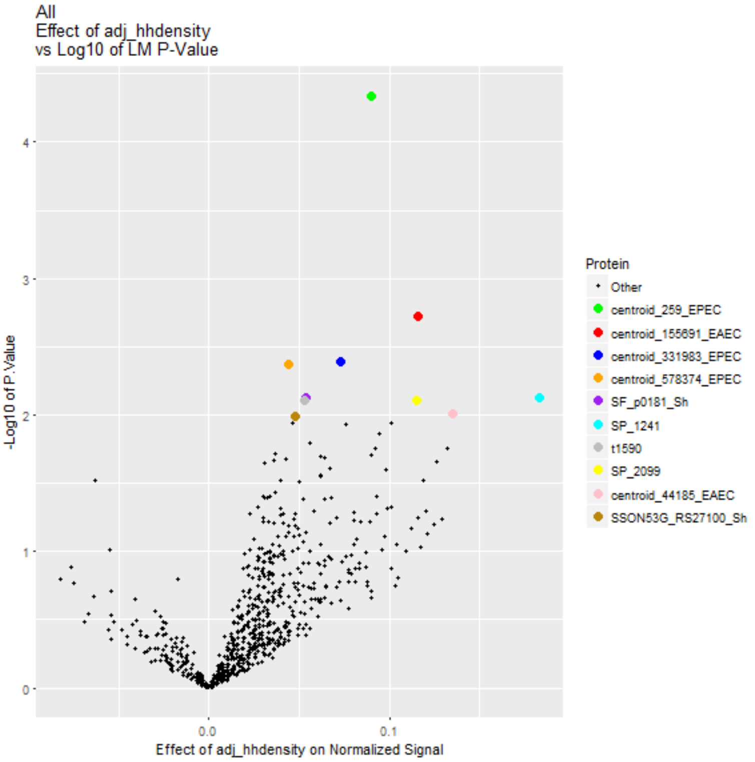

Figure S9. Biological and environmental factors associated with IgG specificity

A. Mother's age

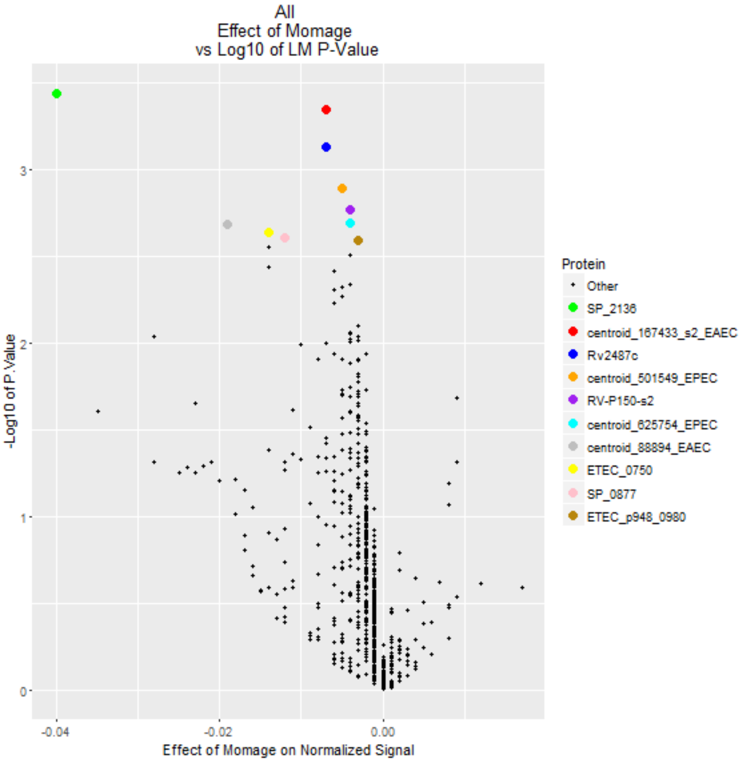

B. Infant sex

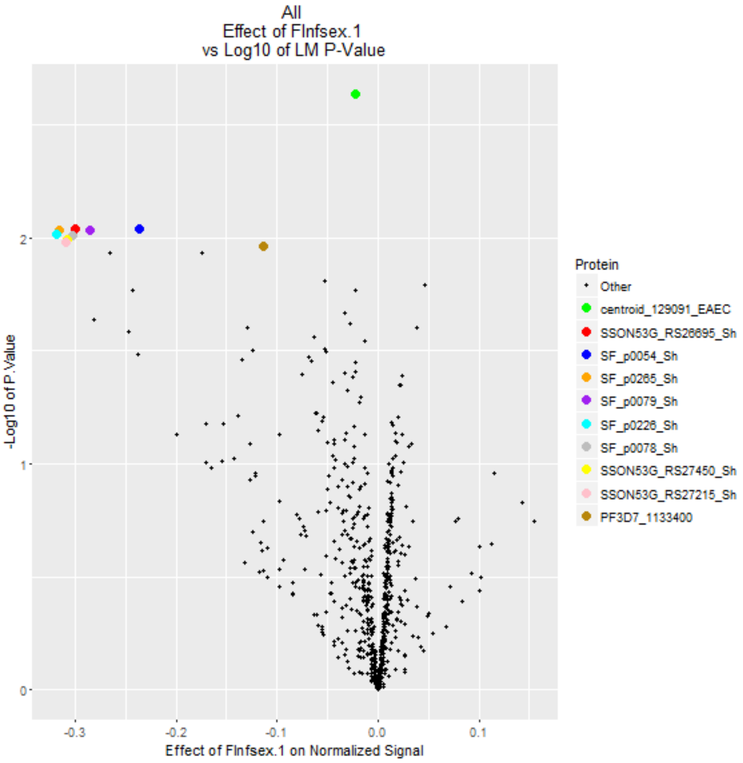

C. Time post-partum

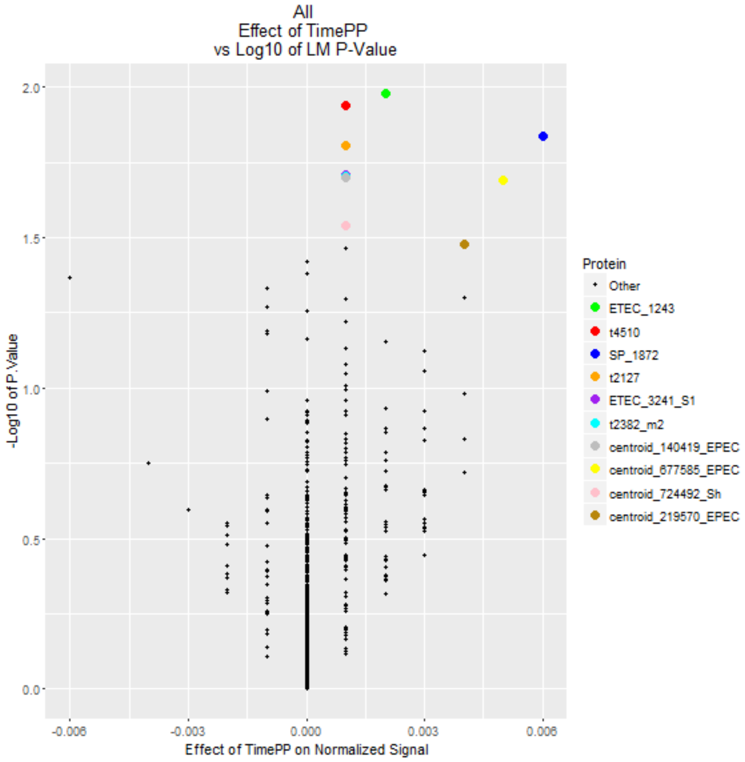

D. Parity

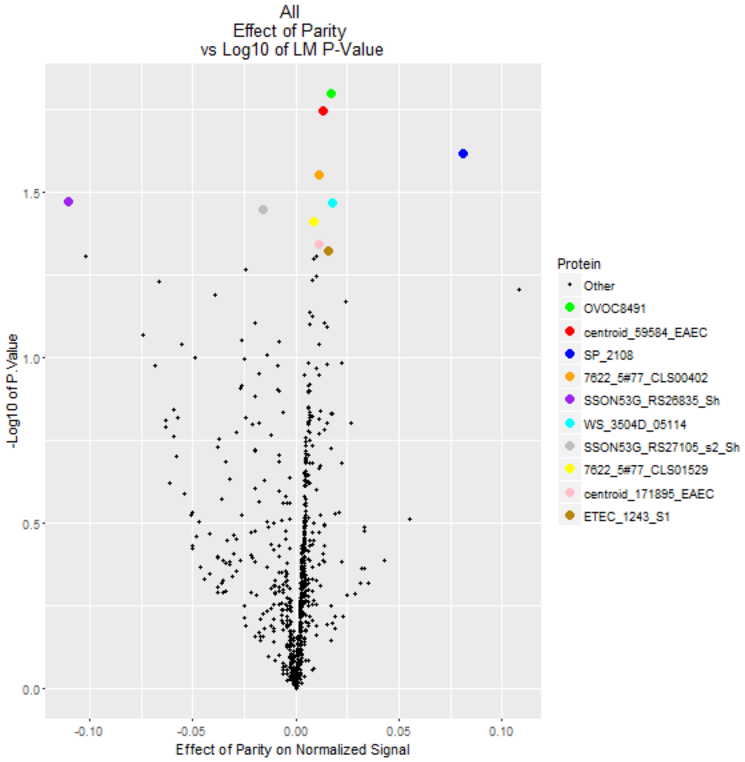

E. Cesarean delivery

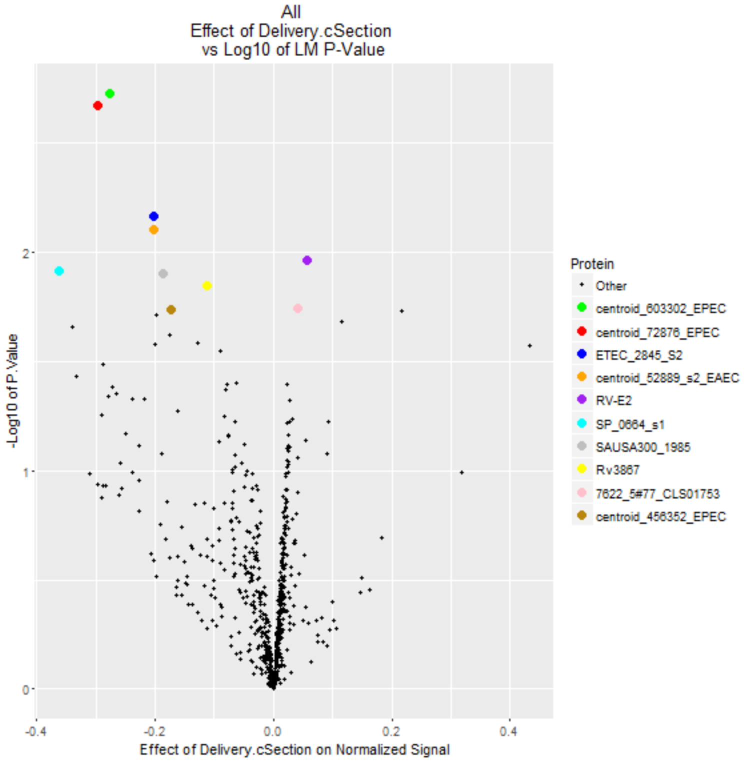

F. BMI code: underweight

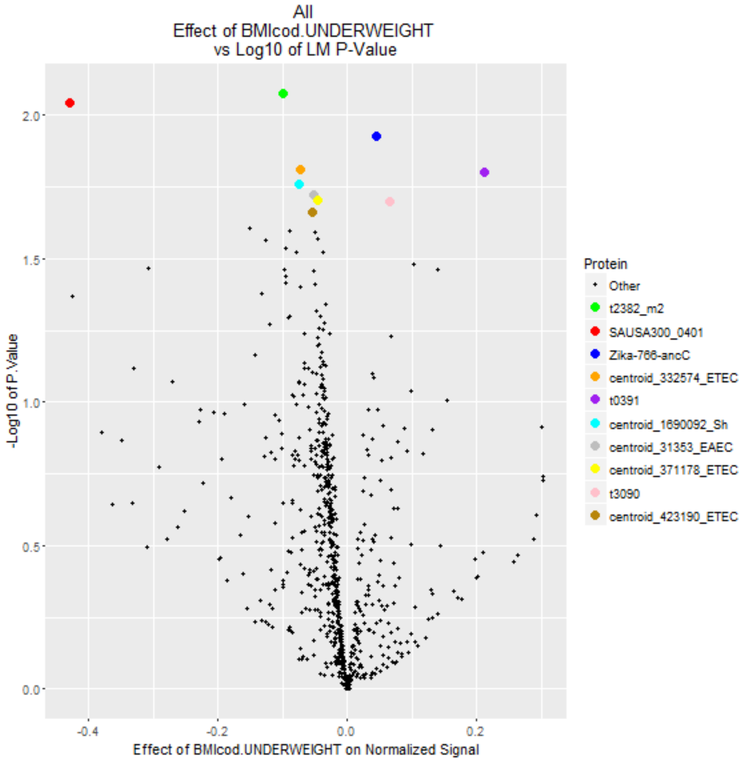

G. BMI code: overweight

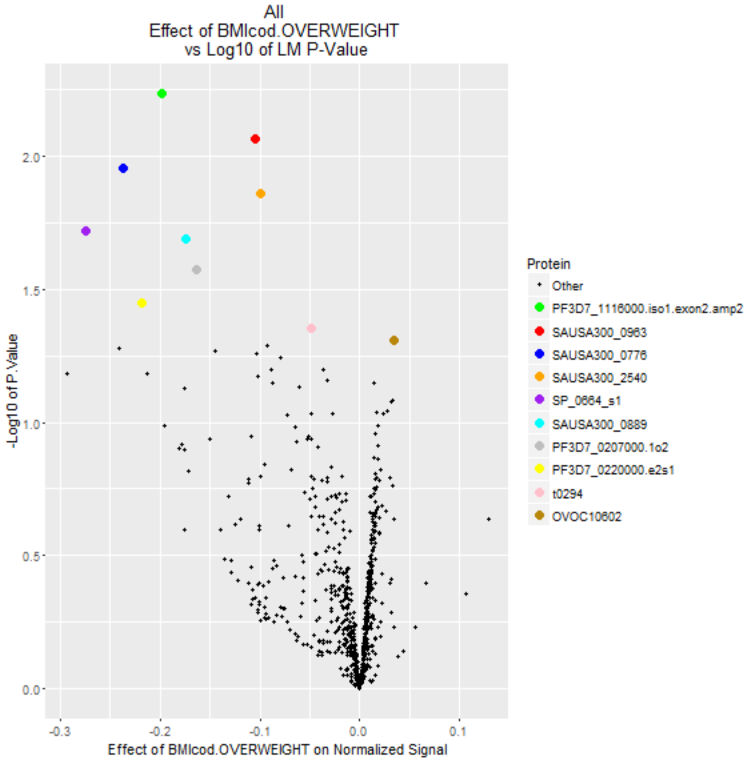

H. BMI code: obesity

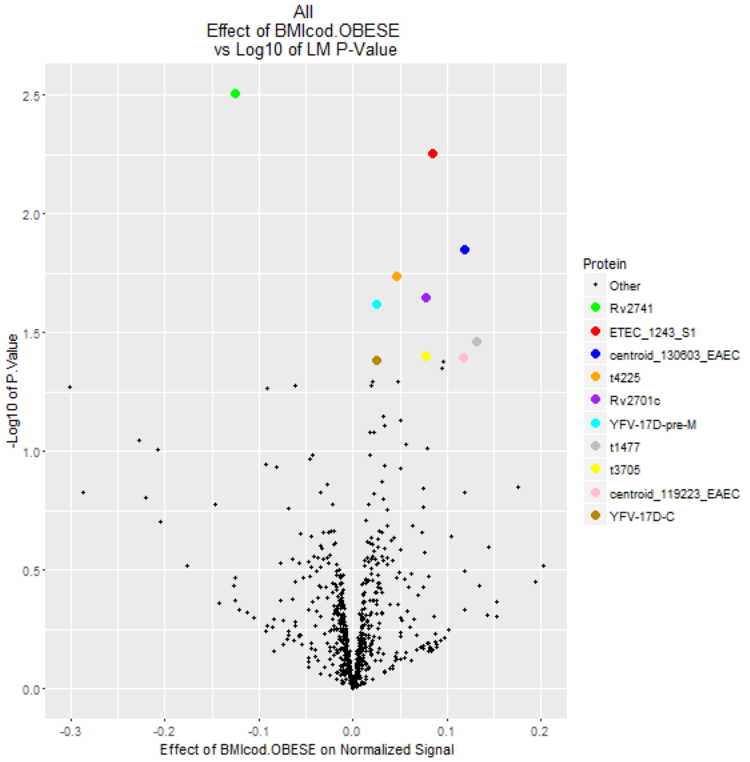

I. Mother’s height

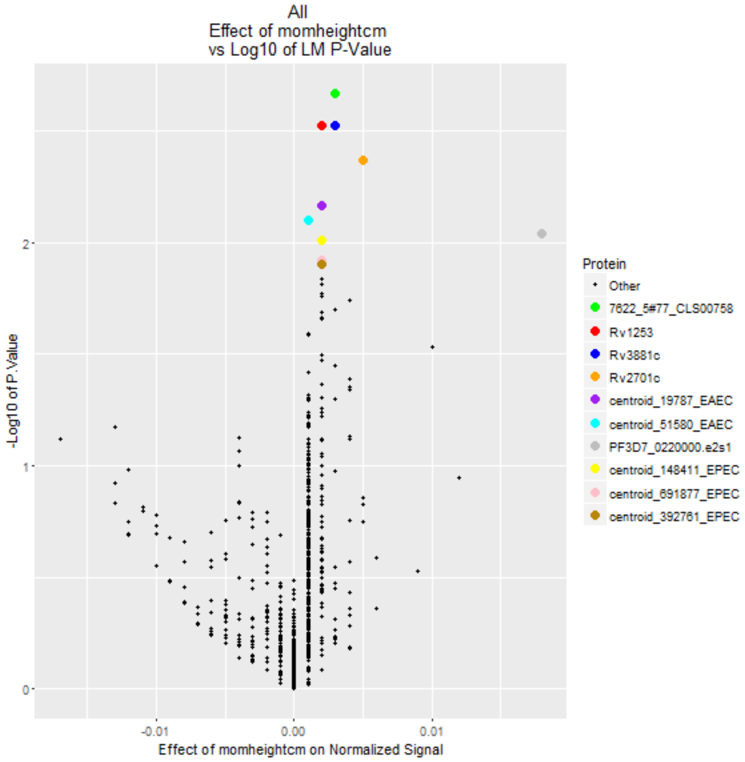

J. Companion animals in household

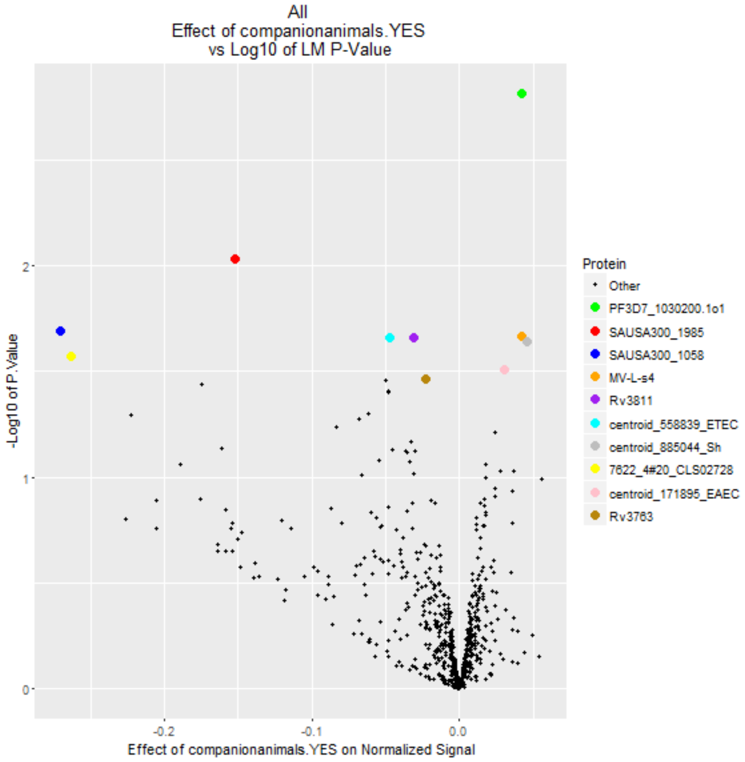

K. Agricultural animals

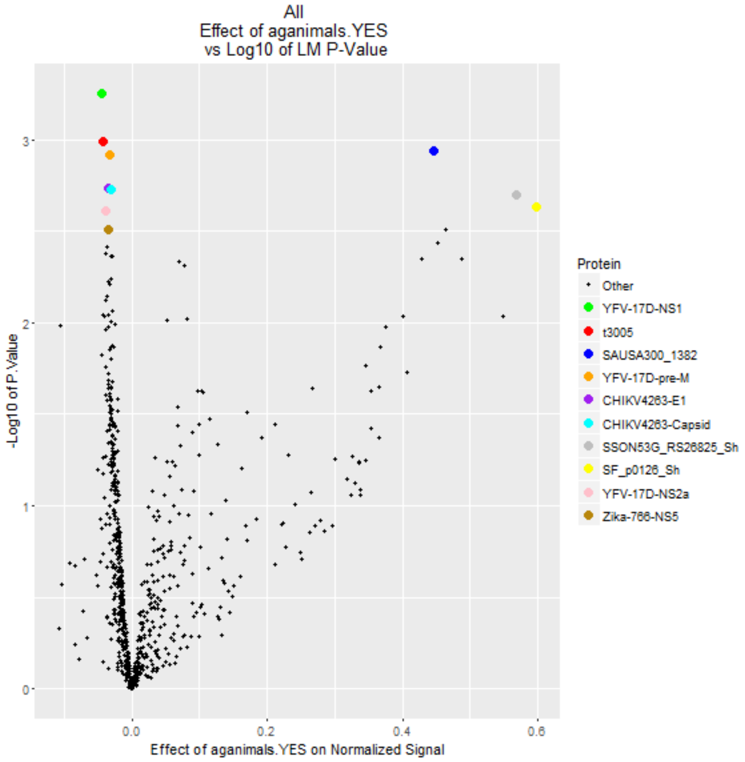

L. Household density

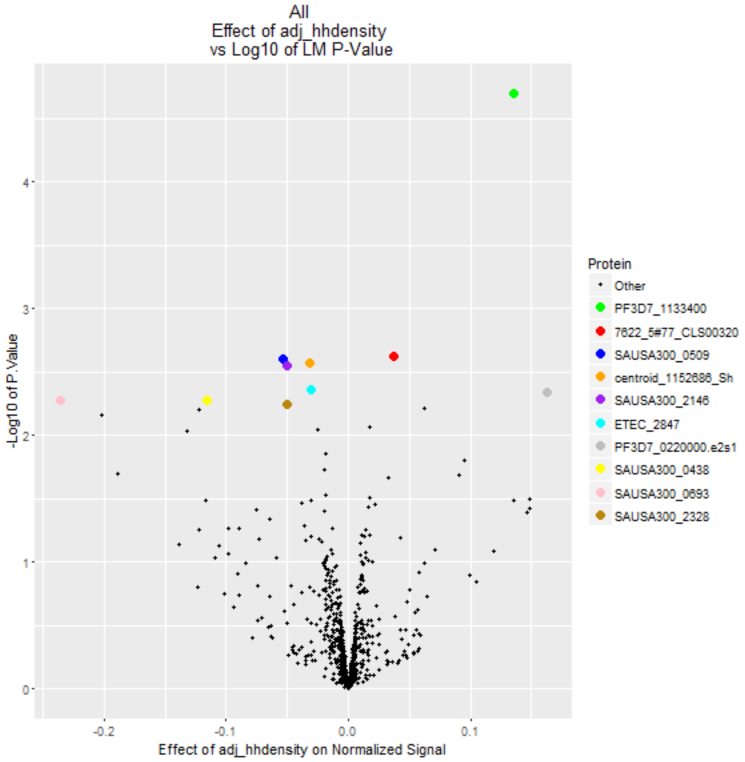

Supplement: Supplementary file 1 [file DataSheet_1.pdf]
